# Llamas (*Llama glama)* enhance proglacial ecosystem development in Cordillera Blanca, Peru

**DOI:** 10.1038/s41598-023-41458-x

**Published:** 2023-09-24

**Authors:** Anaïs Zimmer, Timothy Beach, Sebastián Riva Regalado, Jean Salcedo Aliaga, Rolando Cruz Encarnación, Fabien Anthelme

**Affiliations:** 1 Department of Geography and the Environment, University of Texas at Austin, Austin, TX USA; 2 Laboratorio de Florística, Departamento de Dicotiledóneas, Museo de Historia Natural, Universidad Nacional Mayor de San Marcos, Lima, Peru; 3 Departamento de Etnobotánica y Botánica Económica, Museo de Historia Natural. Universidad Nacional Mayor de San Marcos, Lima, Peru; 4 Área de Evaluación de Glaciares y Lagunas, Autoridad Nacional del Agua, Huaraz, Peru; 5 Universidad Nacional Santiago Antúnez de Mayolo, Huaraz, Peru; 6 AMAP, Univ Montpellier, IRD, CIRAD, CNRS, INRAE, Montpellier, France

**Keywords:** Biogeography, Climate-change ecology, Conservation biology, Biogeography, Climate-change ecology, Conservation biology, Restoration ecology, Ecology, Ecology, Environmental sciences

## Abstract

Worldwide, mountain glaciers are shrinking rapidly. Consequently, large areas are becoming available for the development of novel alpine ecosystems. These harsh environments, however, delay primary succession. In this study with a local community, we conducted an inclusion experiment to investigate whether *Llama glama* influences soils and vegetation primary succession following glacial retreat. At the foot of the Uruashraju glacier in the Cordillera Blanca, Peru (~ 4680 m.a.s.l.), we established four llama inclusion plots and four control plots that we studied from 2019 to 2022, 24–40 years after deglacierization. After three years, the llama plots had significantly increased soil organic carbon and soil nitrogen. In the llama plots, we found a large, significant increase in vascular plant cover (+ 57%) between the second and third years of experimentation, and we identified four new species that were not present in 2019. Our results suggest that *Llama glama*, through their latrine behavior and role as a seed disperser, enhances the primary succession and novel ecosystem formation in recently deglacierized landscapes. Our study provides scientific support that rewilding of native Andean camelids may favor adaptation to glacier retreat and inform conservation and management strategies in proglacial landscapes.

## Introduction

Glaciers are retreating rapidly, and 49 to 83% of glaciers worldwide (excluding ice sheets) will disappear by 2100^1^. The opening glacier forelands provide an opportunity for novel alpine ecosystems to develop. Here, we investigate to what extent llamas may influence soils and vegetation primary succession following glacial retreat and, thus, influence novel proglacial ecosystem formation on a timescale relevant to the rapid pace of anthropogenic climate change.

Since the beginning of the twentieth century, much work has been done on primary succession in proglacial environments^2^, including recent key advances on biotic colonization and pedogenesis^3,4^. The main ecological constraints to plant succession after glacier retreat include dispersal limitation^2^, deficit in facilitators^5^, and abiotic stress from low temperature, low soil moisture, and low soil fertility^6^. In general, the first colonizers are wind-dispersed, while animal- or water-dispersed individuals are underrepresented^7^. Nevertheless, high dispersal capacity alone does not ensure successful succession. Indeed, the very first colonizers after glacial retreat may be opportunistic species that disappear when water and temperature change as the glacier recedes^8^. Thus, a dispersal lag and an establishment lag constrain primary succession^9^. Nurse species are mostly absent during early succession^5^, which limits facilitation when other species need these positive interactions most to overcome extreme environmental stress (stress-gradient hypothesis^10^). Establishing and organizing novel alpine plant communities in proglacial landscapes is therefore complex and slow, and studying primary succession after glacier retreat necessitates long-term observation and experimentation^4^. Manipulative experiments offer great potential for understanding the role of biotic and abiotic factors in driving seed dispersal and plant establishment during primary succession^11^. Notably, in situ experiments might inform us about how external drivers (e.g., livestock grazing, wildlife re-introduction, and tourism) can transform primary succession trajectories.

Crucial to understanding primary vegetation succession is identifying the drivers of proglacial pedogenesis. First, proglacial pedogenesis is slow: skeletic or lithic Leptosols require decades to form and more distinct soil horizons may take hundreds of years^12^. Proglacial soil formation rates vary depending on topography and morphodynamics^13^, climate factors^14,^ parent materials^15^, time^13^, and organisms (i.e., plant-soil interactions^16^, microbial interactions^17^, and biological soil crusts^18^). Proglacial soils are usually phosphorus and nitrogen limited^6^. Although microorganism colonization is key during soil development, harsh proglacial microclimates and nutrient limitations impede microbial activity^6^. Plant colonization is tightly linked to many soil characteristics (e.g., texture, structure, depth, organic fraction, and nitrogen^19^).

Herbivory in glacier forelands might also affect primary succession. Experimental studies have reported a positive short-term effect (i.e., 7–10 years) of herbivory on plant community assembly and ecosystem functioning on bare landslide surfaces (with *Trichosurus vulpecula*^20^) and on Taiga river floodplains (with *Alces alces*^21^). First, herbivory affected plant species composition that, in turn, accelerated the successional trajectory. In addition, browsing decreased the biomass of the dominant species and therefore provided greater opportunities for colonization and access to nutrients by other species (e.g., nitrogen-fixing species). The effects of herbivory on plants, however, has a complex periodicity in which biomass increases in seven to ten years but there is a negative impact on the biomass of the colonizing pioneer species from one to three years^20^.

Second, mammal herbivores enrich soil N and C and soil microbial communities because their wastes increase plant biomass, especially of nitrogen-fixing species^20,21^. Mammal herbivores also feed upon and transport mycorrihzal fungi^22^. For example, interactions between fungi and large ungulates facilitated primary succession at Mount St Helens^23^. Long term (> 30 years) ungulate herbivory can lead to the invasion of unpalatable species, but it can also increase the presence of highly palatable species^24^. In addition, mammals might play a role in exo- and endo- seed dispersion^25^. Mammals found in polar and alpine environments, including muskox, lemming, fox, and ermine are potentially significant dispersal vectors in glacier forelands^18^. Finally, trampling can also affect vegetation positively or negatively by improving seedling recruitment by pressing seeds into the uppermost soil layer^26^ or by increasing lichen clonal growth through fragmentation and spreading processes^27^.

In Andean alpine proglacial environments, Andean camelid dung piles might create more favorable conditions such as resources-rich substrates, sites for seedling establishment, and seed sources, for vegetation establishment (*Vicugna vicugna*^28^, *Lama guanicoe*^29^). Reider and Schmidt^28^ suggested that vicuña dung piles shortcut a 100 + year lag between glacier retreat and primary succession in the Cordillera Vilcanota, Peru. Other studies in different ecosystems have highlighted soil nutrient accumulation by alpaca^30^ or vicuña^31^ dung piles.

Besides soil nutrient enrichment, trampling by large ungulates might also influence soil formation positively or negatively by creating biogeomorphologic changes. First, trampling intensity affects mycorrhizae diversity, which affects soil fertility^32^. Second, trampling can favor pedogenesis by incorporating litter into the soil, enhancing microbial processes and likely influencing the fate of litter carbon, soil organic carbon, and nitrogen mineralization^33^. Third, by causing bioturbation, trampling can expose more mineral soil to erosion and generate finer particles^34^. Moreover, herbivore trampling can compact soils, which might affect soil infiltration, erosion, and nutrient mineralization (e.g., in arid environment^35^). Nevertheless, camelid—versus cattle— trampling creates less compaction^36^ and can improve ecosystem functioning through water redistribution (e.g., in hilly dryland^37^).

Globally, experimental approaches to study pedogenesis and vegetation primary succession after glacier retreat are crucially missing. A few studies implemented experimental research designs in glacier forelands, which mainly included seed burial^38^ and experimental warming^39^. Moreover, all studies took place in the European Alps, while pressing ecological challenges related to deglacierization exist globally^40^. While glaciers melt worldwide, emerging proglacial ecosystems are becoming important components of the freshwater and carbon cycles. They might also act as sediment sinks that buffer upstream hazards and be refugia for cold-adapted species^41^. The establishment and organization of novel proglacial systems is a key concern for adaptation to glacier retreat. Thus far, we have no study that includes experiments with large herbivores, including camelids, in deglacierized landscapes. However, experimental approaches are necessary to understand more precisely the modalities of proglacial ecosystem development and favor adaptation^4^.

In this study, we used an inclusion experiment to understand if the presence (i.e., herbivory and trampling effects) of llamas (*Llama glama*) in glacier forelands can enhance primary succession after glacier retreat. We evaluated the effects of llamas on (1) soil pedogenesis and (2) primary vegetation succession in a proglacial ecosystem of the Tropical Peruvian Andes, where glaciers are exceptionally vulnerable to global warming^42^. First, we hypothesized that the llama dung piles would drive soil enrichment, catalyzing soil formation processes; soils would have higher nitrogen and carbon contents in the presence of llamas. Second, we hypothesized that the presence of llamas enhances the primary succession of the vegetation. With llamas, we expected an increase in plant biomass, as well as higher nitrogen and phosphorus foliar contents because of soil enrichment. Finally, we hypothesized that the llamas would function as seed transporters between lowlands, highlands, and valleys, by carrying seeds in their hooves, wool (epizoochory), or stomach, and stimulate seed germination via endozoochory. Our study aims to replicate a natural phenomenon to enhance ecosystem formation and inform conservation and/or land management strategies in proglacial landscapes.

## Materials and methods

### Study area

**Figure 1:**
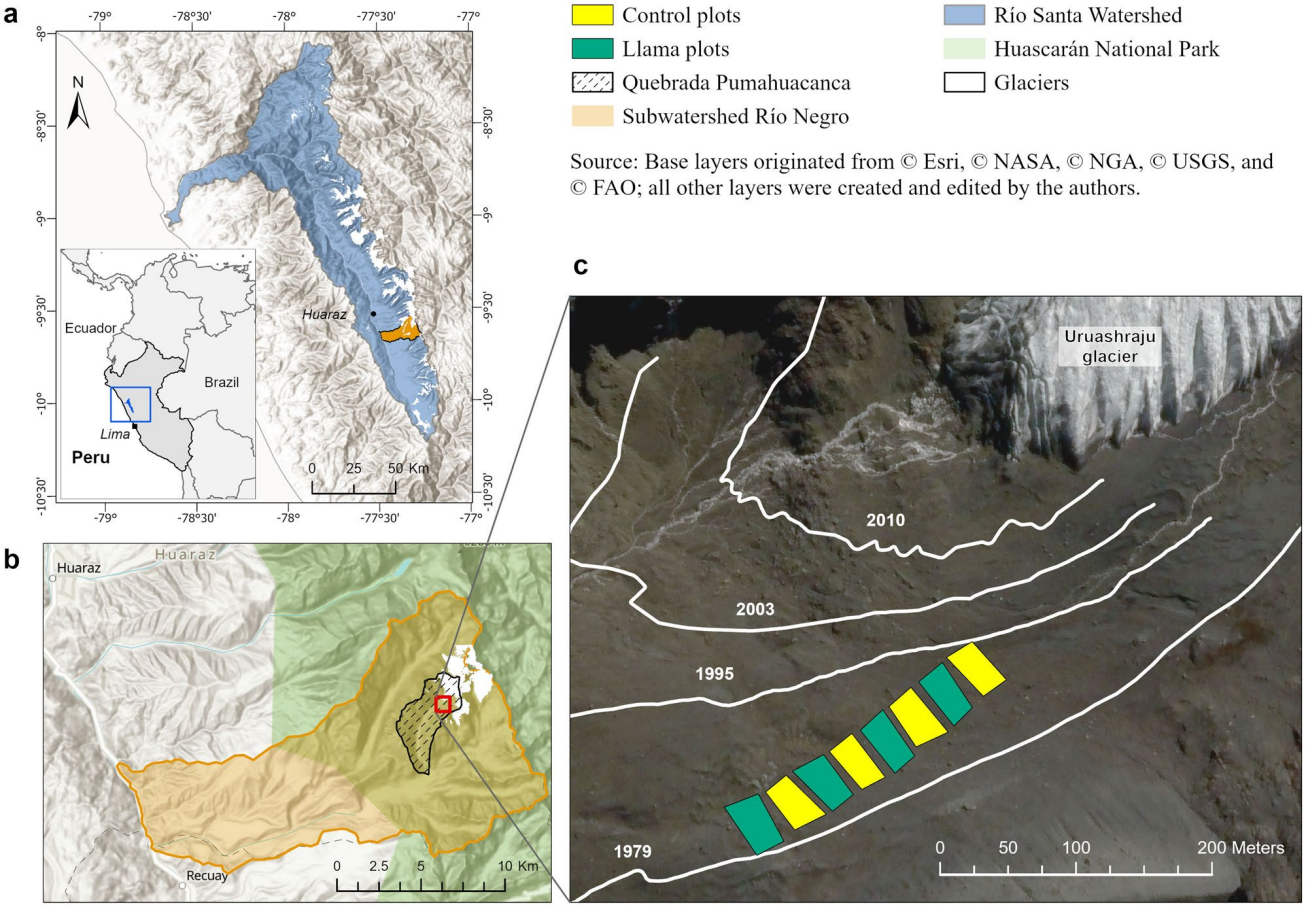
Location and study site set-up. Map of location with respect to the Santa River watershed (**a**), and Río Negro sub-watershed (**b**). Map of the experiment within the Uruashraju glacier foreland (**c**). The glacier retreat outlines were produced and provided by the ANA (Área de Evaluación de Glaciares y Lagunas, Autoridad Nacional del Agua, Huaraz) based on topographic field surveys of the glacier fronts since 1948, and analysis of photographs. Maps generated by authors with licensed software ArcGIS Pro 3.0.2 (https://www.esri.com/en-us/arcgis/products/arcgis-pro/).

We conducted our experiment in the Uruashraju glacier foreland located in the Quebrada Pumahuacanca within the National Park of Huascarán (NPH), Cordillera Blanca, northwestern Peru (Fig. 1). The Cordillera Blanca is the world's most extensively glacier-covered tropical mountain range^43^. In the 1960s, the imminent extinction of the overhunted and emblematic vicuña, a native Andean camelid, prompted the creation of the NPH. This area is semi-arid and has highly seasonal precipitation with 80% of the 700 mm (~ 3500 m.a.s.l.) to 1000 mm (~ 4550 m.a.s.l.) per year falling between October and May^44^. The mean annual air temperature at 3450 m.a.s.l. is approximately 12–14 °C with strong diurnal variability^45^ but little seasonal variability due to its tropical location (9°S). The geology of the Quebrada Pumahuacanca is dominated by granodiorite and tonalite, with outcrops of the meta-sedimentary Jurassic Chicama formation, that consists of shale, pyritic siltstone, and quartzite with volcanics in some areas^46^. Sulfide-rich lithologies occur within the glacier foreland and their oxidation after the retreat of the ice is linked to low pH values^47^.

The Pumahuacanca Quebrada (from 4050 to 5605 m.a.s.l.) has patches of high Andean woodlands (e.g., *Polylepis spp.* and *Gynoxis spp.)*, wetland ‘*bofedales*’ dominated by cushion-forming plant communities (e.g., *Distichia muscoides*), and grasslands dominated by tussock grass such as *Cinnagrostis rigida*^48^*.* Certain species like *Werneria nubigena* indicates the presence of non-native livestock and overgrazing. Within the NPH, Pasture User Committees (CUP) use these high pasture ecosystems to graze their livestock. This is the case of the CUP of Arwaycancha in the Quebrada Pumahuanca. In recent years, the uncontrolled introduction of exotic livestock for agropastoralism and tourism activity has caused degradation of pastures and the disappearance of some native species within the Park^48^. Due to their soft footsteps and prehensile split upper lips Andean camelid minimize impact on the groundcover compared with introduced livestock^49,50^. The NPH has thus supported camelid breeding initiatives as an ecosystem and biodiversity conservation strategy^51^.

The study site (77° 19′ 20" W, 9° 35′ 47" S) lies between 4680 and 4700 m.a.s.l. in a terrain deglacierized between 1979 and 1995^52^ (Fig. 1). This research was in collaboration with the NPH and the *Llama 2000 Asociación*, a local community of farmers, who own the llamas*.* The farmers’ own village of Canrey Chico, Olleros has contaminated water due to acid rock drainage (ARD) that threatens their agriculture^53^. As an adaptation strategy to glacier retreat, the farmers aim to find solutions to increase the vegetation cover in recently deglacierized areas in order to reduce ARD. Previous floristic plot surveys carried on in the Uruashraju glacier foreland identified 47 vascular plant species (Zimmer et al., *unpublished data*; Table S3). We selected the Uruashraju foreland based on the *Llama 2000 Asociación*’s shared interest in our research questions, the presence of llamas in the adjacent Quebrada, data availability for deglacierization chronology, floristic comparisons within the overall glacier foreland (Table S3), and the ecological representativeness of the site. Considering the socio-geoecological aspects of the region, we expect our results to be generalizable to the overall Cordillera Blanca and, possibly, to other mountain ranges.

### Context and experimental design

The *Llama 2000 Asociación* launched the *Llama 2000* Project to increase the value of local llama breeding and conserve an essential part of their Inca biocultural heritage. In 2019, the community aimed to strengthen its activity promoting the sustainable management of llamas and vicuñas to develop community-based tourism along the Qhapaq Ñan trail, enhance local economy, and develop climate change adaptation strategies. We collaborated with the *Llama 2000* community and the NPH to test a novel adaptation strategy to glacier retreat by introducing llamas in the Uruashraju glacier foreland. Manipulative experiments better predict the causal impacts of short-term ecological change and allow for a better control of confounding factors^54^, which here included livestock disturbance, llama stocking rate and visit frequency to the site, and camelid movement between Quebradas. Here, we are testing a novel adaptation strategy to glacier retreat by introducing llamas in a glacier foreland in a region where the density of Andean camelids has strongly decreased over the last century and where camelid breeding initiatives have been strengthened by National Entities (i.e., NPH and SERNANP—Servicio Nacional de Áreas Naturales Protegidas por el Estado^51^).

**Figure 2:**
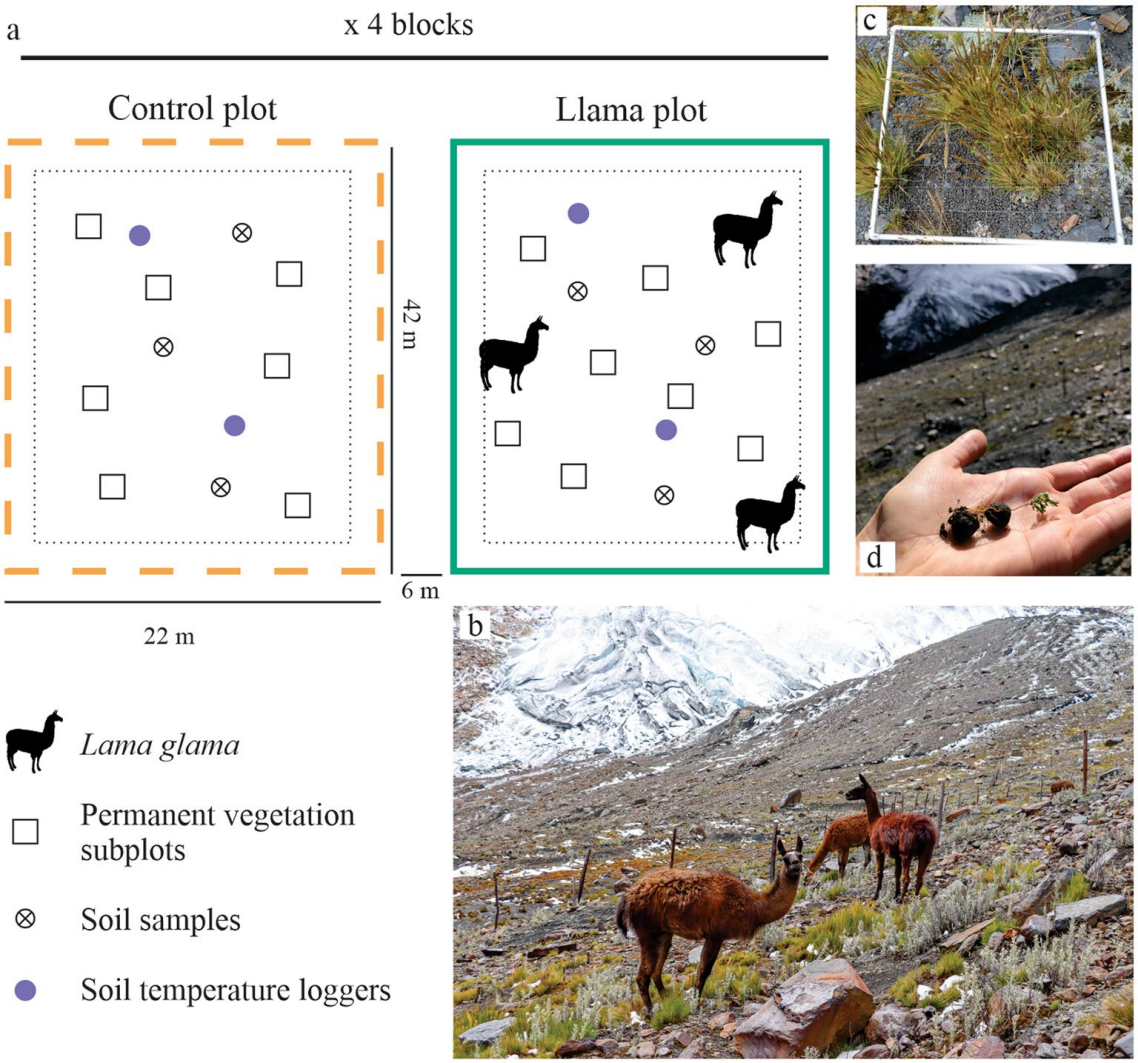
Experimental design and in situ surveys. Design of the experiment (**a**), *Llama glama* within a llama plot (**b**), 1m^2^ vegetation subplot (**c**), seedling germinated from llama feces found within the experiment in June 2022 (**d**).

In June 2019 we set up a complete block design with four monitoring blocks—i.e., four llama plots and four control plots (Fig. 2a, b)—across a chronosequence since deglacierization: the south corner of the experiment was ice-free in ~ 1979 and the north corner in 1995 (Fig. 1c). We built eight plots (925 m^2^ each) over 12,500 m^2^ of low to moderate slopes. Within each plot, we randomly established eight (1m^2^) permanent subplots (Fig. 2c). Thanks to personal communications with the *Llama 2000* farmers, specialists of the NPH, and a local zootechnician, we determined the animal load and grazing intensity within the plots based on (1) the natural density of vicuña observed in proglacial landscapes in the Cordillera Blanca (NPH, *Unpublished data*), (2) llama and vicuña behavior (herd grouping and displacement between altitudinal range), and (3) vegetation cover of the study site and llama nutritional requirements. These considerations allowed us to replicate a natural phenomenon (i.e., several proglacial landscapes have been identified as vicuña habitat within the NPH). From June 2019 and to 2022, in each fenced plot, three llamas grazed for three days each month (cf. Appendix 1 – Experimental design).

### Field data collection

To test the effects of llamas—grazing, trampling, and latrine behavior—on proglacial ecosystem development following our three hypotheses (i.e., soil enrichment, vegetation succession, and seed dispersal), we collected: (1) pedological, (2) plant diversity and productivity data, and (3) llama dung pile samples. The data collection took place at different times (Table 1).

**Table 1:** Data collected during the field evaluations of the experiment.

Data collected	May 2019 (dry season)	December 2020 (wet season)	May 2021 (dry season)	June 2022 (dry season)
Floristic & geomorphic surveys	X	X	X	X
Plant functional traits	X			X
Soil sampling	X			X
Llama dung pile sampling				X

First, in May 2019, before introducing the llamas, we conducted the first floristic and geomorphic evaluations of the 64 subplots (i.e., Hypothesis 2). At each subplot, we identified all the vascular species (Table S4). We estimated visually their relative surface cover, density, necromass, and their average height. As well, we recorded the presence of biological soil crusts (BSC) measuring their heights and relative covers. In each subplot, we also estimated soil granulometry —percentage cover of sand (< 2 mm), gravel (< 2 cm), rock (< 10 cm), and block (> 10 cm). Every year from 2019 to 2022, we replicated the same biotic and abiotic data collection for each of the 64 subplots to study the effects of the presence of llamas over time. In addition, in 2020, 2021, and 2022 we visually assigned a greenness index (0, 1, or 2) to each subplot based on the proportion of green vegetation within each subplot. During the field evaluation in 2020 and 2021 we observed the presence of eaten pasture within the llama plots, which could have biased our estimations of plant cover and height. To overcome this concern, in 2022 we waited three weeks between the last grazing treatment and our field evaluation, giving time for the plants to grow again after the grazing event. We also reported eaten pasture within the control subplots in 2020 and 2021, indicating either that the llamas escaped at least once from the fences, despite attentive care, or the llamas grazed within the control area during their arrival on site while the farmers were distributing the animals between plots.

In addition, we measured three plant functional traits: leaf dry matter content (LDMC), total nitrogen (N), and phosphorus (P) to study plant productivity (i.e., Hypothesis 2). Plant leaf N is directly related to photosynthesis and respiration and reflects the trade-off between greater photosynthetic capacity and potentially suffering more herbivory^55^. LDMC correlates negatively with potential relative growth rate and positively with leaf lifespan^56^. Leaves with high LDMC tend to be relatively tough and are thus assumed to be more resistant to physical hazards (e.g., herbivory) than are leaves with low LDMC^55^. Therefore, LDMC is an indicator of a plant’s resource use strategy: usually, stress tolerant and slow growing species found in stressed environments display high LDMC and low N, whereas low LDMC and N-rich leaf are characteristic of competitors and ruderals with fast resource acquisition strategies^55^. For the trait measurements, we selected four species based on (1) their presence in the 8 plots (Table S4), (2) their potential as ecosystem engineers (i.e., *Pernettya prostrata*: slope stabilization; *pers. obs.*, *Senecio sublutescens*: high litter production and micro-habitat creation; *pers. obs.*), and (3) their palatability (*Agrostis. tolucensis; P. prostrata*; *pers. obs*.).

In June 2022, we collected 10 leaf samples from different individuals of the same species within each plot. Each sample is a composite of a minimum of three individuals, summing to approximately 15 to 40 leaves per sample depending on the species. We used stratified sampling within the four llama plots, collecting half of the 10 samples from individuals located inside or down-slope of a dung pile and the other five samples from individuals located three meters outside of dung piles (cf. Appendix 1 – Field data collection). The collection of plant material and soil samples was conducted in accordance with relevant recommendations and authorizations of the SERNANP (licenses N°008–2019-SERNANP-JEF and N°005–2022-SERNANP-JEF). The soil samples were exported to the USA under the USDA (United States Department of Agriculture) permit to receive soil P330-17–00,299 and P330-20-00161_20200706. All methods were carried out in accordance with relevant guidelines. As importantly, because we measured and counted the same plant specimens present within the permanent subplots in the control and the llama plots every year, we did not collect the full plant specimens; the collection of plant specimen would have been destructive for the experiment and biased the floristic evaluations results for this study but also for the long-term monitoring of the experiment. Therefore, the plant species identified and studied have not been deposited in a public herbarium, since the registration process requires the full specimen (including vegetative material and root). Accordingly, the plant identification was done in the field by botanists from the Museum of Natural History of the National University of San Marcos (UNMSM), Lima, Peru (Sebastián Riva and Jean Salcedo). Regarding the measurement of the plant traits (nitrogen, LDMC, phosphorus), we only collected the minimum amount necessary for the analysis, collecting only the leaves directly from the plants in the field, and trying to be as minimally destructive as possible for the experiment.

Then, to infer the effect of the llamas on soil development (i.e., Hypothesis 1) in May 2019 (before introducing the llamas) and June 2022 (three years after initiation), we collected three soil samples distributed in the upper, middle, and lower slopes of each llama plot. Each sample is itself a composite of three subsamples collected within 1m^2^, between 3 to 15 cm deep. We excluded from the sampling any pure organic surface layer, litter, and vegetation (cf. Appendix 1 – Field data collection).

Additionally, we collected data of covariate variables that might potentially affect our response variables: subplot slope degree and soil temperature. To measure soil temperature, we buried two HOBO 8 K Pendant data loggers (five to 10 cm depth) per plot within the upper and lower parts of the plot. Since June 2019, the 24 data loggers have recorded temperature every four hours. The slope and soil temperature measurements allowed us to account for confounding factors such as a slope effect on soil composition (i.e., Hypothesis 1) or vegetation cover (i.e., Hypothesis 2) or an effect of the proximity of the glacier on soil temperature. We used the available precipitation and air temperature data produced by the ANA (Autoridad Nacional del Agua, Huaraz, Peru) from a weather station installed within the Uruashraju glacier foreland) to corroborate our soil temperature analysis and infer the role of seasonality on the response variables (Figure S2).

Finally, to estimate the fertilization effects of the llamas on the soil composition and their role as seed dispersers (i.e., Hypothesis 3; Fig. 2d), we collected three fecal samples from three different llama dung piles in each llama plot. The Environmental Quality Laboratory (EQL) in Huaraz, Peru analyzed samples for nutrients, and the Museum of Natural History of the UNMSM in Lima, Peru analyzed seeds in the samples (cf. Appendix 1 – Field data collection).

### Laboratory and herbarium method

#### Laboratory analysis

To infer the role of llamas on pedogenesis, we analyzed soil pH, particle size distribution, soil organic (SOC) and inorganic (IC) carbon, soil nitrogen (N), and carbon isotopes (δ^13^C). We analyzed the 48 soil samples at the University of Texas at Austin, USA, mostly at the Soils and Geoarchaeology Laboratory. Soil pH was determined using a soil:solution ratio of 1:2. Because estimating SOC in proglacial soil is complex (due to low SOC and carbonate or clay contents), we used two different methods: sequential Loss On Ignition (LOI) and Elemental Analyzer (EA). First, we analyzed the 48 samples from 2019 and 2022 using the LOI method for SOC and IC (cf. Appendix 1 – Laboratory analysis). Second, for the 24 samples collected in 2022, we measured SOC and ^13^C by isotope ratio mass spectrometry. Values of δ^13^C provide information about the origin of the SOC. Bardgett et al.^57^ linked high δ^13^C values to the presence of ancient carbon, and Kielland and Bryant^21^ used δ^13^C values to examine the effects of herbivory on soil carbon composition. We measured N using an EA CHNS-O. Finally, at the Laboratory for Environmental Archaeology of UT Austin, we performed Particle Size Analysis (PSA) after deflocculation (cf. Appendix 1 – Laboratory analysis).

The EQL in Huaraz, Peru measured leaf LDMC, N, and P, as well as the total organic C, total N, and total P of the fecal samples. We measured LDMC only for *P. prostrata* and *S. sublutescens* since we did not expect major changes within the tussock grasses^58^. N and P were measured for all species. At UNMSM in Lima, we evaluated the viability of the seeds using the Tetrazolium test (Table S22; cf. Appendix 1 – Laboratory analysis).

### Data analysis

Data analyses were performed using the statistical software R version 4.2.2. To test our first hypothesis (i.e., llamas catalyze soil formation)**,** we first computed summary statistics for all the soil response variables (texture, pH, SOC, IC, N, and δ^13^C) for the control and llama plots. Afterward, we used linear models to test the significance of the variation assignable to the llama treatment. We included as response variables texture, pH, SOC, IC, N, and δ^13^C. We used a fixed blocking factor (4 blocks) to control for time constant unobserved effects within each block (Fig. 2), and “Treatment” and “Years” as fixed factors. We first ran a model based on the experiment’s full three-years (including samples from 2019 and 2022). Second, we ran two separate models, one for the 2019 data and one for the 2022, to remove the variable “Years” from the model to gain degrees of freedom and reduce standard errors. Since the soil nitrogen data from 2019 and 2022 are not comparable because of different drying methods, we did not run the full 3-years model on soil nitrogen. We report the results of all models within the results section and supplementary material.

To test our second hypothesis, we assessed plant community changes based on species richness—count of species per subplot to estimate the taxonomic diversity of the subplots, plant cover, and plant functional traits (i.e., LDMC, N and P, plant height, necromass, and greenness) between the control and llama plots. Plant height and necromass were community weighted for each subplot. As well, we calculated a percentage change in plant cover between 2019 and 2022. First, we computed summary statistics. Then, as for the soil data, we used linear models with a fixed blocking factor to analyze the effect of llamas on plant cover, height, and necromass. The linear models for the vegetation response variables included “Treatment”, “Years”, and “Block” as explanatory variables. In addition, the initial plant cover values reported in 2019, were used in the plant cover analysis as a co-variate to increase the power of the test. We computed the total percentage cover of each species for the overall experiment to select the four species most representative of the plant community. Then we ran distinct models for the four species to test for the effects of the llamas on the species selected. We used Generalized Linear Models with a Poisson distribution to test the effects of the llamas on plant richness and greenness because both variables were discrete, and we hypothesized that species richness patterns were scattered during the first stage of primary succession (i.e., species not aggregated). To evaluate differences between years and between control and llama treatment, when relevant (i.e., Plant cover and Greenness), we computed pairwise comparisons using the *emmeans* package with Bonferroni corrections (*emmeans* function). Afterward, we used linear regressions to assess the effect of the initial subplot cover on the percentage change in cover between 2019 and 2022 and the 2022 plant cover. We log transformed the variable Plant Cover to increase the fit of the model (Shapiro–Wilk normality test: p-value = 0.094, W = 0.988; Breusch-Pagan test: p-value = 0.46; Chi-square = 0.547). We computed all the linear and generalized linear models using the *lm* and *glm* functions of the *stats* library and the model fit diagnostics were checked using the *DHARMa* package.

To analyze variation in soil temperature we used the period with complete data from 23.06.2019 to 22.05.2021. We calculated summary statistics and used Wilcoxon tests to assess the difference between control and llama treatments. To infer differences in air temperature that might have affected our response variables we compared mean daily temperatures over three periods corresponding to our field evaluations dates: June 2018 to May 2019; June 2019 to May 2021, and June 2021 to May 2022. We used ANOVA and Tukey Honestly Significant Difference tests to assess differences between the three periods. Finally, we tested for significant differences in slope gradient and granulometry of the subplots between the llama and control plots with T-tests and Wilcoxon tests (*ggpubr* library).

## Results

### Environmental characteristics of the plots

In 2019, prior to llama manipulation, there was no significant difference between the mean slope of the control and llama plots. Similarly, there was no significant difference in sand, gravel, rock, and block contents between the llama and control plots (Table S5). Between June 2019 and May 2021, we observed lower daily minimum (− 0.45 °C; *p-value* = 3.8e-10) and higher daily maximum temperatures (+ 3.88 °C; *p-value* = 0.001) within the llama plots than within the controls (Table S5). The air temperature data (from June 2018 to May 2022) showed significantly lower temperatures between June 2021 and May 2022 than between June 2018 and May 2019 (diff = − 0.22, p-value = 0.02), and between June 2019 and May 2021 (diff = − 0.31, p-value < 0.001; Figure S2; Table S2). The mean daily precipitations between 2017 and 2020 displayed differences in precipitation between the dry season (June to September) and the wet season (October to May)^44^.

### Effect of llama presence of soil development

**Figure 3:**
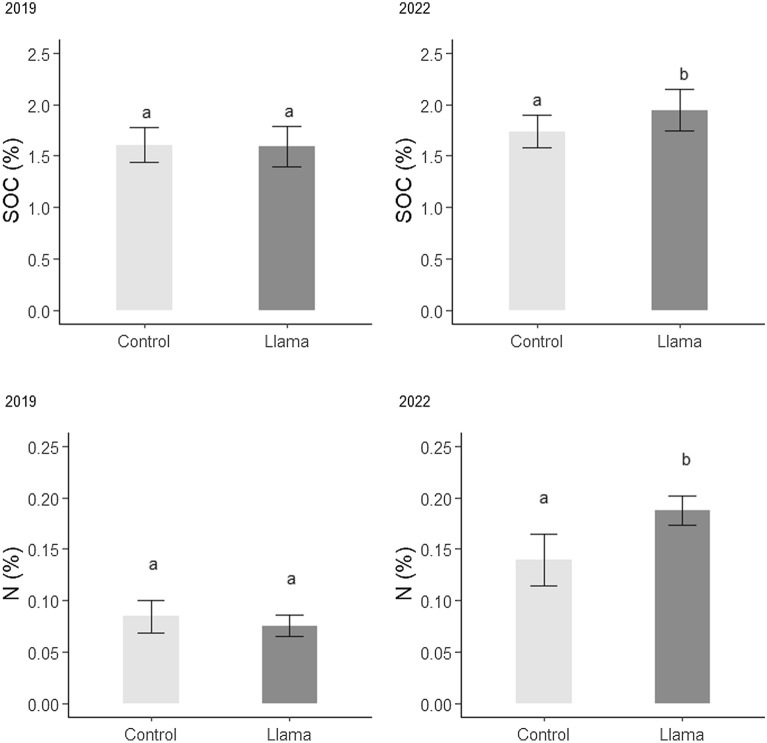
Effects of llamas on soil organic carbon (SOC by LOI) and nitrogen (N) (95% confidence intervals) in 2019 and 2022. Means not sharing any letter are significantly different by Bonferroni tests (p ≤ 0.05).

After 3 years of treatment, we observed an effect of the llamas on the silt and sand contents (Figure S3). Both the full three-years model (2019 and 2022 data) and the 2022 model showed an increase in silt within the llama plots (respectively *p-value* = 0.017; Table S7 and *p-value* = 0.059; Table 2). Only the full three-years model showed a significant decrease in sand within the llama plots (*p-value* = 0.019; Table S7). Between 2019 and 2022, SOC and IC obtained by the LOI method and N were affected positively by the llamas (respectively + 0.2% SOC, *p-value* = 0.047; + 0.1% IC, *p-value* = 0.047 and + 0.05% N, *p-value* < 0.001; Fig. 3, Table 2, and Table S6). The SOC data from the EA method also showed a significant effect of the llamas on the SOC in 2022 (+ 0.16% SOC, *p-value* = 0.047). The δ^13^C signature of SOC in 2022 did not differ significantly between the treatments as well as pH (Table 2). The organic C of the dung pile (15.4%) was approximately 9 times higher than the SOC of the control plots in 2022 (1.74%). Similarly, dung pile total N (5.07%) was 36 times higher than the N in the 2022 control plots (0.14%; Table S11). We did not detect a leaching effect from the upslope to the downslope portions of the experimental plots, because there was no significant difference in soil composition between the samples position on slope.

**Table 2:** Significance of the effects of the llamas on soil properties in 2019 and 2022: texture (clay, silt, and sand), pH, organic carbon (SOC) by LOI and EA methods, inorganic carbon (IC), nitrogen (N), and δ13C.

	2019				2022			
	Est	SE	t value	Pr(> t)	Est	SE	t value	Pr(> t)
Clay	4.498	2.084	2.158	0.044 *	0.601	2.511	0.239	0.813
Silt	1.945	1.379	1.411	0.174	5.406	2.696	2.005	0.059
Sand	− 6.441	2.939	− 2.191	0.041 *	− 5.995	4.450	− 1.347	0.194
SOC by LOI	− 0.015	0.094	− 0.155	0.878	0.205	0.096	2.128	0.047 *
SOC by EA					0.158	0.075	2.122	0.047 *
δ13C					− 0.233	0.136	− 1.716	0.102
N by EA	− 0.009	0.008	− 1.158	0.261	0.048	0.011	4.385	< 0.001 ***
pH	0.093	0.069	1.354	0.192	0.250	0.163	1.535	0.141
IC	− 0.007	0.044	− 0.155	0.878	0.097	0.045	2.128	0.047 *

Bold values indicate significance (p ≤ 0.05). The full coefficients of the models are reported in Table S8 and S9 in Supplementary material.

### Primary succession of vegetation

#### Vegetation biomass

**Figure 4:**
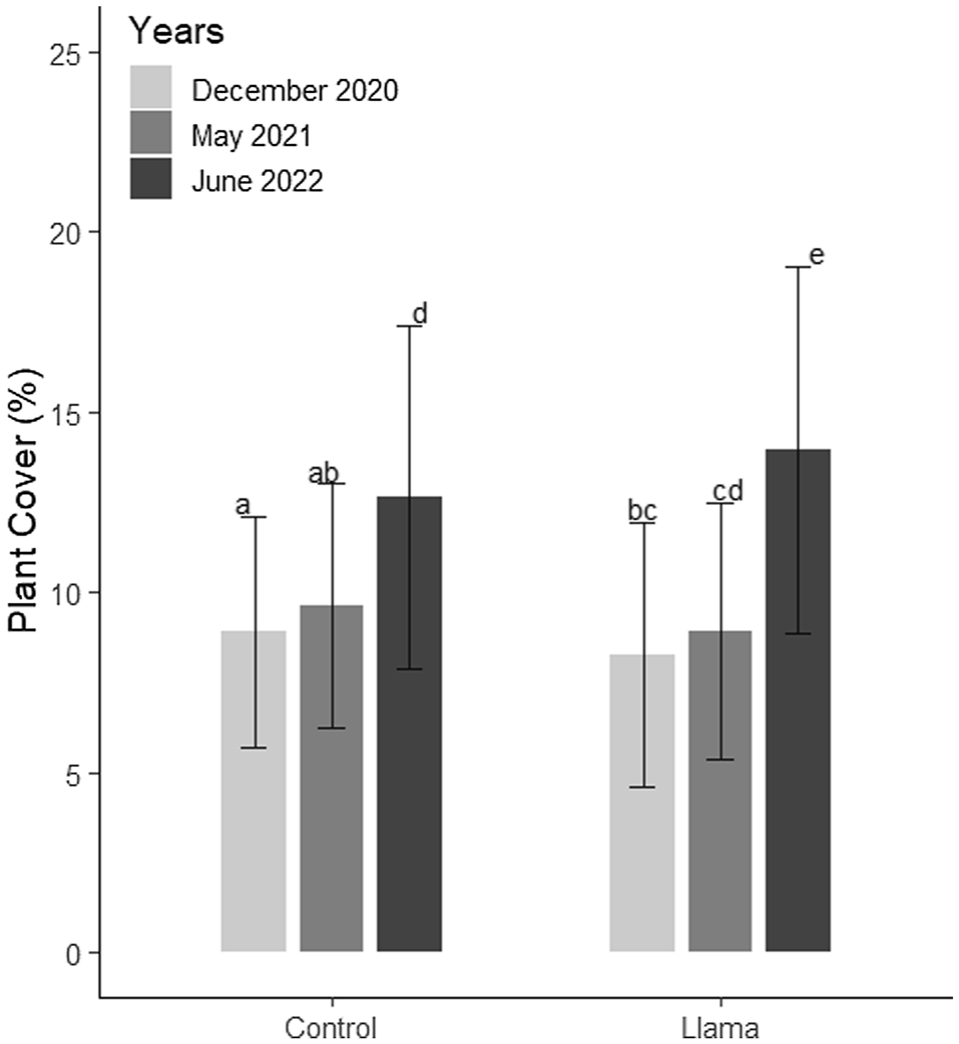
Effects of llamas on plant cover (95% confidence intervals). Letters represent the significantly different groups according to the post hoc contrasts (p ≤ 0.05).

In 2019, the mean subplot cover of the llama (7.96%) and control (11.38%) plots were not significantly different (*p-value* = 0.12; Table S12). Between 2019 and 2022, the presence of llamas had a significant (*p-value* < 0.001) positive effect on plant cover (Table S13). Between 2020 and 2021, both the llama (*p-value* = 1) and the control (*p-value* = 1) plots did not show significant increases in vegetation cover (Fig. 4; Table S14). In contrast, from 2021 to 2022, the mean cover in the llama plots increased from 8.91% to 13.97% (+ 57%, *p-value* < 0.001; Fig. 4; Table S12 and S14). Linear regressions to model the relationship between the percentage change in cover between 2019 and 2022 and the 2019 subplot cover showed that the control subplots with higher cover in 2019 have gained less cover during the three years than the subplots with lower cover in June 2019 (*p-value* = 0.004, R-squared = 0.24). The trend is lowered within the llama subplots (*p-value* = 0.018, R-squared = 0.18; Figure S4). We did not detect a significant effect of the llamas on the BSC.

Across the four surveys of the experiment, we observed a total of 14 plant species. Of these, 13 were identified at the species level (Table S4). Results from the generalized linear model showed no effect of the llama treatment on species richness between 2019 and 2022 (*p-value* = 0.534), whereas there was a significant effect of the year 2022 (*p-value* = 0.015; Table S13). Nevertheless, the *Belloa piptolepsis*, *Oritrophium limnophilum*, *Senecio rufescens,* and an unidentified juvenile individual (*sp.*) appeared only in the llama plots in 2022. Also, *Melpomene peruviana* appeared only within the control plots in 2022 but was already present in 2019 (Table S4). The four species with the highest total cover were *Cinnagrostis rigida*, *Senecio sublutescens*, *Minoides kunthiana,* and *Agrostis toluscensis* (Table S4). Linear models ran separately for the four species showed that the llama presence had a higher effect on the plant covers of *S. sublutescens* (β = 0.45; *p-value* < 0.001) and *M. kunthiana* (β = 0.36; *p-value* = 0.01) than on *A. toluscensis* (β = 0.11; ns) and C. rigida β = 0.12; *p-value* = 0.02) (Table S16; Figure S5).

#### Plant traits

**Figure 5:**
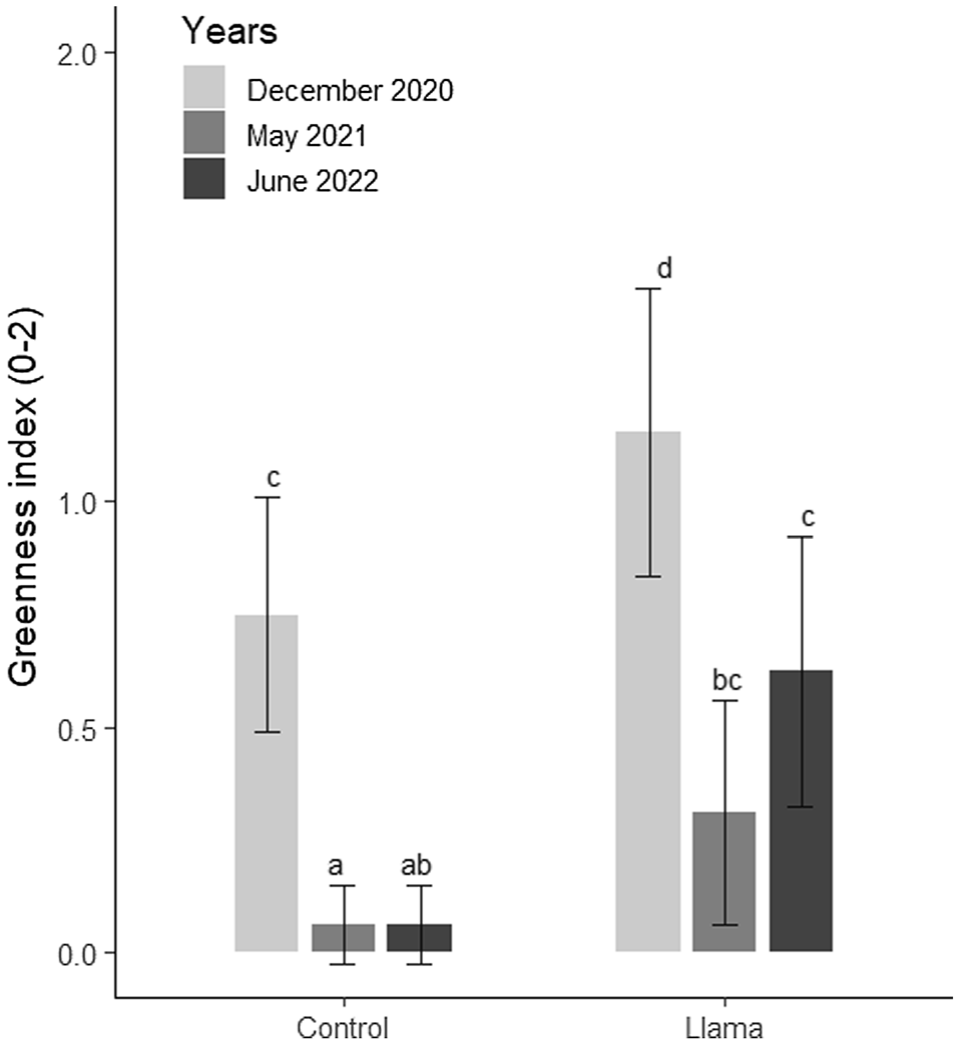
Effects of llamas on greenery (95% confidence intervals). Letters represent the significantly different groups according to the post hoc contrasts (p ≤ 0.05). Note: December is the wet season.

Plant traits analysis revealed that there is no effect of the llamas on the LDMC of *Pernettya prostrata (p-value* = 0.06). However, we observed higher LDMC within the samples collected within the llama plots but far from dung piles (i.e., Llama: no dung pile) than the samples collected in the control plots (+ 28.7 mg.g^-1^) and samples with dung pile influence (+ 32 mg.g^-1^; Figure S6; Table S17). There is a positive effect of the llamas on the LDMC when there is no dung pile influence (*p-value* = 0.016; Table S18). We did not observe significant changes in LDMC, N, and P for the other species sampled. Subplot greenness was positively affected by the llamas (Table S13). There were significant decreases in plant greenness between 2020 and 2021 in the control plots (*p-value* ≤ 0.001) and the llama plots (*p-value* ≤ 0.001; Fig. 5; Table S15). Although there was no significant increase in greenness between 2021 and 2022 for both treatments, we observed higher greenness index within the llama plots than within the control plots in 2021 (*p-value* = 0.002) and in 2022 (*p-value* = 0.002; Table S15). During the four floristic surveys we have reported several observations of pasture eaten within the subplots in both the control and llama plots (Table S20). We did not detect an effect of the llamas on plant necromass and height.

### Seed and germination potential

**Figure 6:**
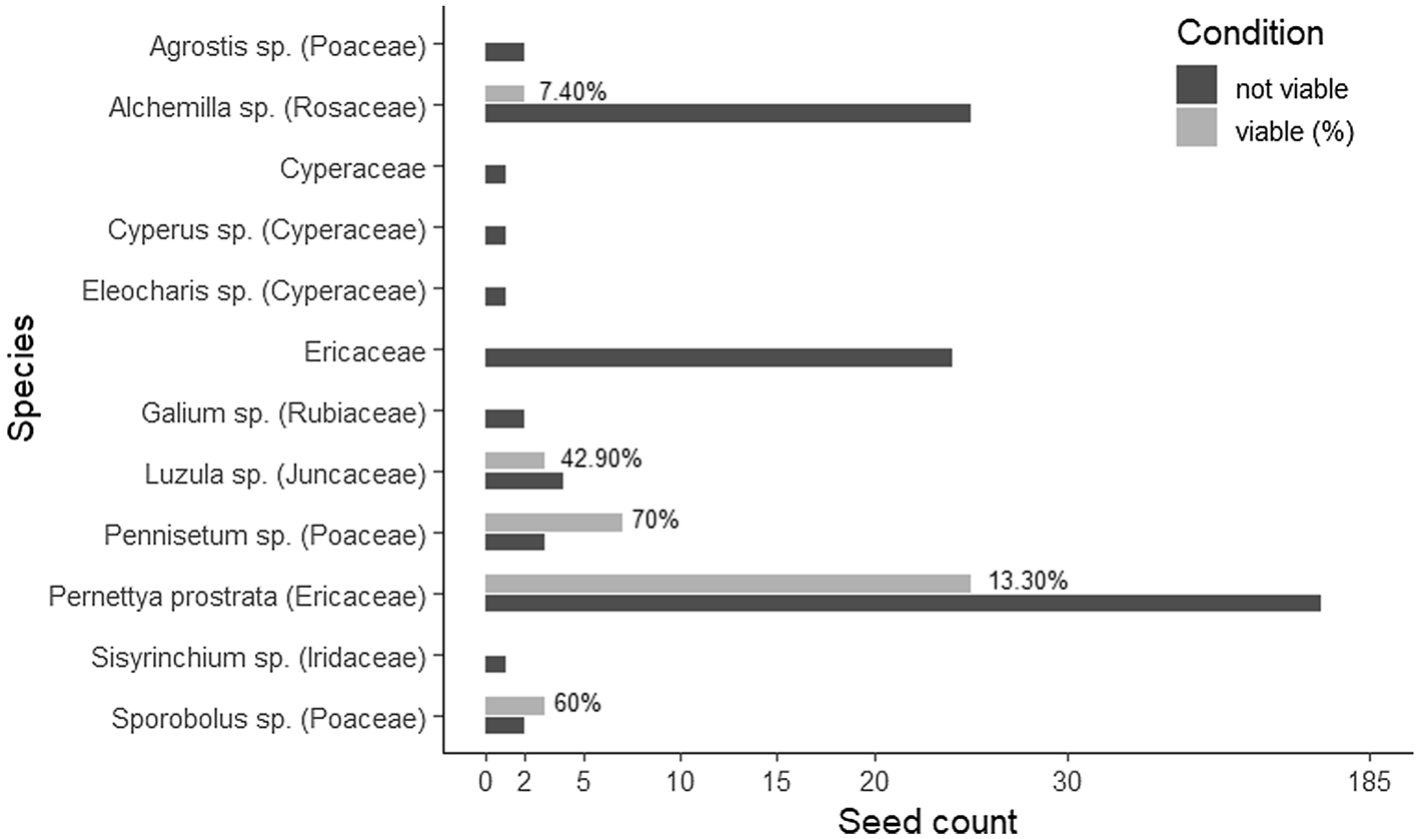
Seed viability per species found within the llama pellets (n = 266). The percentages expressed are the percentages of viable seeds for each species. For the taxa identified at the species or genus level family names are in brackets.

In total, 266 seeds were found within the llama fecal pellets’ samples (Fig. 6). Seeds of 12 plant species belonging to seven families were identified within the pellets, from which two families (Poacea and Ericaceae) were also present within the llama plots (Table S21 and S22). The viability tests showed that five of the seed species identified still maintain their germinative power between 7.4% and 70% (Fig. 6; Table S21).

## Discussion

Our study investigates the effects of llamas on pedogenesis and vegetation primary succession after glacier retreat. We expected that the presence of llamas and their latrine behavior would catalyze soil development and enhance vegetation succession. Unlike previous studies on camelid herbivory in proglacial landscapes, our results demonstrate that in addition to drive soil enrichment and increase plant cover, the llamas have transported viable seeds via endozoochory to the proglacial landscape, likely facilitating dispersal processes.

The analysis of the soil samples showed that over three years, llamas had a positive impact on proglacial pedogenesis through a modification of the soil texture and increases in SOC and N. First, the very high SOC, N, and P measured in the dung pile samples demonstrate that Andean camelid dung piles act as nutrient hotspots within the proglacial landscape^29^. Albeit we do not disregard limitations due to the lower temporal resolution of the dung pile analysis, the increase in SOC and N observed in the llama plot soil samples are likely due to a translocation of nutrients via surface runoff from the dung piles to the adjacent areas^30^. Andean camelids graze in dung pile areas because of their nutritional benefits^28,31^. Therefore, based on our results we hypothesize that in a longer term there would be also an intra-systems redistribution of the nutrients within the proglacial pasture from old to new dung pile sites, in addition to the transfer from downstream to proglacial systems. Besides dung pile nutrient addition, camelid trampling might also facilitate litter and BSC incorporation to the subsurface soil, accelerating its decomposition and favoring C and N enrichment^34^.

Soil texture can serve as proxy for proglacial pedogenesis. Proglacial chronosequence studies have reported a reduction of the fraction of sand and an increase in silt with increasing age^12,59^. We suggest that here the observed increase in silt is due to llama bioturbation processes^34^ that favor silt incorporation. Second, the increase in plant cover would have reduced erosion processes and increased the capture of recent, carbonate-rich sediment within the denser vegetation, creating feedback effects of the vegetation on soil development (i.e., biogeomorphic interactions^60^). These texture changes reveal an accelerated soil development with the llamas, which would lead to changes in several soil characteristics and microtopography changes, and affect the spatial redistribution of water and soil resources, as well as proglacial ecosystem functioning and productivity^37,59^. Future analysis will investigate nitrogen isotope signature and microbial activity (e.g., mycorrhizae), to improve our understanding of how the llamas affect belowground communities and therefore the overall nutrient cycles in recently deglacierized landscapes.

The δ^13^C signatures of the llama and control plots are consistent with previous observations in glacier forelands and demonstrate the presence of ancient carbon^57^. The slight depletion (but not significant, *p-value* = 0.1) in δ^13^C between the control and llama plots could support an enrichment in modern carbon in line with the observed increase in SOC. Indeed, δ^13^C is higher in low-carbon soils and decreases as SOC increases due to recent organic carbon inputs^14^.

After three years, the presence of llamas had a substantial impact on the primary vegetation succession at the Uruashraju glacier foreland, in line with hypothesis 2. The optimization of aboveground net primary production by mammals is reported in grass and shrubland systems^24,61^, and few studies also reported positive pathways in arctic tundra^62,63^. Here, the increased plant cover and overall greenness of the fenced plots imply that the llamas have increased the development of the aboveground vegetation. Although we reported significantly lower minimal and higher maximal soil temperatures within the llama plots in comparison to the control plots, we do not consider that these environmental variations would significantly affect plant growth. The differences are relatively small and occur at temperature ranges that are not critical to alpine plant life^19^. Similarly, we recognize a potential effect of the decreased air temperature between 2018 and 2022, but those differences are relatively small to have a significant effect on vegetation development. We do, however, suggest that differences in precipitations caused the increase in cover observed within the control plots between May 2021 and June 2022. Similarly, we hypothesize that the high greenness value observed in December 2020 in comparison with the May 2021 and June 2022 is due to the highest precipitation occurring during the wet season. Further work will continue to monitor soil temperature and precipitations to infer the role of the llamas on micro climatic characteristics and decipher the role of climatic covariate variables during primary succession.

These changes are likely to have significant effects on the landscape evolution, enhancing vegetation-soil feedback cycles^59,64^. For example, *Senecio sublutescens* had the highest cover increase in the llama plots and is very likely to produce significant quantities of litter that will be redistributed to the soil surface through llama trampling and litter decomposition^33^. Thus, the effects of the llama latrine behavior on soil pedogenesis will lead to increased plant cover^29^.The higher llama effects on the plant covers of *S. sublutescens* and *Minoide kunthiana* in comparison to *Agrostis toluscensis* and *Cinnagrostis rigida*, suggests that species abundance might be related to species palatability. Although there is scant literature on these species, *C. rigida* and *A. toluscensis* are known to be palatable for Andean camelid, whereas *Senecio* species are generally not very palatable to livestock^65^, and *M. kunthiana* has a very low stature that might be difficult for llama grazing. Albeit the effects of the llamas on plant cover do remain low after three years, we reported significant changes within the proglacial ecosystem during this short period whereas proglacial ecological dynamics are known to be slow^2^. Indeed, most of experimental studies are carried out for longer periods (i.e., 10 to 35 + years)^20,21^. Thus, our study indicates that biotic and abiotic modifications caused by the presence of the llamas can quickly have significant effects on pedogenesis and primary succession after glacier retreat. Our short-term results and the low temporal resolution of our sampling underline the need to study both short term and long-term effects of camelid herbivory on novel proglacial ecosystems.

Llamas have modified the functional traits of the proglacial plant community. Studies show that plant greenness, leaf thickness, and LDMC are tightly linked to plant growth, chlorophyll production, and correlated with leaf N^66,67^. The increase in subplot greenness within the llama plots suggests higher photosynthetic activity and plant growth, which is highly likely connected to the dung pile soil nutrient enrichment. In addition, the higher LDMC values of *P. prostrata* observed within the “llama—no dung pile influence” group in comparison to the “controls” suggests a defense mechanism of *P. prostrata* responding to a grazing effect^68^. The individuals of *P. prostrata* suffering from grazing and not beneficiating from the enriched soil may have lowered their growth rate and invested more resources in structural protection of the photosynthetic tissues, as an adaptation to avoid the effect of defoliation (i.e., avoidance strategy^68^). While the lower values found within the llama plots with “dung pile influence” suggest a nutrient enrichment effect from the dung piles and therefore a reverse response of *P. prostrata* LDMC, lowering the negative consequences of the llama grazing (i.e., tolerance strategy). The llama latrine behavior has locally (i.e., a few meters) transformed a low-nutrient system into a more productive system, and individuals benefiting from the enriched substrate have faster regrowth traits^69^. We hypothesize that variation in climatic conditions (precipitation during the wet versus dry season) explain the higher greenness index observed in December 2020 and the strong decrease in greenness between December 2020 and May 2021.

Our results also suggest a decrease in competition between species within the llama plots. The linear model positive coefficient of the 2019 plant cover means that plots with higher cover in 2019 displayed stronger positive effects from the llamas. In the control plots the 2019 initial cover explained a fourth of the variation of the percentage change in cover between 2019 and 2022; thus we hypothesize that in the absence of llamas, the subplots with low cover are more likely to experience higher rates of plant cover increase because of a potentially reduced plant-plant competition. Whereas in the llama plots the strength of the relationship is lower, indicating that other factors (i.e., the llamas) might have reduced competition through the physical effect of grazing^62^. However, we do not exclude the possibility that abiotic factors, such as precipitation or temperature, might also have influenced plant-plant interactions within the experiment. We also hypothesize that the llamas would have an effect on abiotic facilitation though trampling (e.g., alteration of nurse features such as rock), which are keys associations during the first stage of primary succession^5^. The presence of llamas in proglacial landscapes may modify primary succession trajectories and allow plant communities to overcome the harsh environmental conditions.

We did not observe a notable change in plant richness within the experiment. Although, we registered four new species that appeared only within the grazed areas. Similarly, we noted the disappearance of one species within the llama plots in 2022 that was present in 2019. The disappearance of *C. ovata* might be due to the temporal variability in moisture inputs within the proglacial systems, since *C. ovata* is mainly found in wet terrains^70^. The precipitation data available and analyzed did not allow us to test differences in precipitation between years. Long-term monitoring is necessary to fully evaluate the effects of the llamas on plant richness and plant functional traits . Finally, future research will need to investigate the feedbacks between plant defoliation from herbivory and its effect on soil biota and all factors on the interactions of mycorrhizae^71^.

Ungulates are known to act as dispersal agents through endozoochory, carrying seeds on their coats, or between their hooves (epizoochory), or simply spitting out seeds after mastication or rumination^72,73^. Here, we show that llamas have the potential to disperse seeds of different plant families and types from lower elevation or neighboring valleys to the proglacial habitat (Table S18). Within the llama dung we found viable seeds from five distinct species: *Alchemilla sp.*, *Luzula sp.*, *Pennisetum sp.*, *P. prostrata,* and *Sporobulus sp.,* of which only the *Pernettya* and *Luzula* were reported during the floristic surveys of the experiment, indicating an external seed input. The seeds of *Pernettya, Sporobolus,* and *Luzula,* being viable and endozoochore, evidence the role of Andean camelids as seed transporters by endozoochory^74^. Notably, the *Luzula racemosa*, which was not present in 2019 within the experiment but found in 2022 in the llama plots, is present at lower elevation in the valley (57 years after deglacierization; Table S3) and therefore might have been brought to the experiment by the llamas. Since llamas move regularly between vegetation communities for foraging and ranging, both endo- and exozoochorous dispersal might provide an important potential mechanism for colonization. We recognize that different physical and chemical treatments of the seed (e.g., digestive treatment, feces humidity, and deterioration) might have produced different viability results or also impacted seed dormancy and therefore germination. In addition, we hypothesize that seeds evaluated as “not viable” during our viability test from the llama pellets, could also have been transported via llamas exozoochory from the lower ecosystems, and thus be present in viable condition within the experimental area. For example, species such as *Plantago tubulosa* and *Werneria pygmaea—*peat-forming cushion plants^48^*—*and *Alchemilla pinnata*^74^ have been found to be part of the Andean camelid diet, and might be transported via llama endo or exozoochory to the more recently deglacierized terrains. Those species are present within the Uruashraju glacier foreland in terrain deglacierized for more than 57 years (Table S3). In addition, we do not exclude the role of human-directed movement of the llamas and anthropogenic seed dispersion. First, the farmers might have influenced the displacement path and the selection of the pasture area during the daily grazing at lower elevation, which in turn could have affected the identity of the species grazed and the seeds present within the fecal pellets, as well as the seed dispersal by exozoochory. Second, we also recognize the role of humans as dispersal agents from lower elevation to the proglacial area. Finally, we do not exclude the role of the llamas as facilitator of the spread of invasive exotic plants by epizoochoory or endozoochory such as *Rumex acetosella*^75^. In the Andes, the study of archaeobotanical remains have given insights on past landscape uses and on the role of camelid in shaping plant communities over long time scales^76,77^. Here, our results demonstrating that llama dung can be a source of viable seeds in deglacierized terrains provides promise for the role of Andean camelids in shaping the emerging and future proglacial landscapes.

In the Andes, the Puna ecosystem was a primary center of ungulate domestication^49^. However, since the Spanish conquest in the 1500s, European livestock (e.g., cattle, horse, donkey, and sheep) have almost completely replaced domestic camelids and ancestral herding practices. Introduced livestock —often associated with overgrazing—have several negative ecological impacts in Andean ecosystems^49^. First, exotic animals can alter the structure and composition of the native vegetation^75^, the hydrologic soil functions^36^, and can increase erosion by soil compaction and by uprooting plants^49^. Here we showed that the presence of Andean camelid favors pedogenesis and vegetation primary succession that may result in enhanced high Andean alpine biodiversity and novel proglacial ecosystem functioning. By reducing runoff, increasing carbon sequestration, and potentially reducing ARD, the reintroduction or rewilding of native Andean camelid may favor adaptation to glacier retreat while benefiting the local economy. Andean camelids are known for their fiber and meat production, and are also involved in tourism activities and Andean folklore^31^. Rewilding interventions as strategies for biodiversity conservation and ecosystem functioning are novel management alternatives that could be applied to the benefit of proglacial landscapes^78^.

## Conclusion

Our study shows that Andean camelids (*Llama glama*) can enhance soil pedogenesis and primary vegetation succession in glacier forelands. In three years, the latrine behavior of the llamas led to soil organic carbon and nitrogen enrichment and changes in soil texture, suggesting enhanced proglacial pedogenesis pathways. Similarly, we reported a significant increase of the plant cover and the appearance of four species not previously documented. We found that llamas can function as seed transporters bringing seeds from lower elevations or neighboring valleys to the proglacial habitats and possibly facilitating colonization processes, albeit more years might be necessary to also observe an increase in plant richness. These results have at least three implications. First, llamas might help overcome some of the constraints of primary succession after glacier retreat like dispersal limitation, low soil fertility, and plant competition, lowering dispersal and establishment lags^9^ and enhancing ecological connectivity between deglacierized landscapes. Second, our experimental simulation of a natural process suggests that other Andean camelids displaying similar behavior might also enhance proglacial ecosystem establishment (i.e., vicuña, alpaca, and guanaco^29^). Lastly, our findings may have implications for the conservation and management of novel proglacial ecosystems around the world. Locally, our study supports the goal of national and local efforts in re-introducing Andean camelids from local communities. The experimental protocol designed in this study will allow long-term monitoring to continue to assess the changes observed in our three-year study and discover what changes may happen at longer timescales.

### Supplementary Information

Supplementary Information.

## Data Availability

The datasets used and/or analyzed during the current study are available from the corresponding author on reasonable request.
