# Supplementary material for: Llamas (Llama glama) enhance proglacial ecosystem development in Cordillera Blanca, Peru
Source: Sci Rep. 2023 Sep 24;13:15936. doi: 10.1038/s41598-023-41458-x (PMC10518316; doi:10.1038/s41598-023-41458-x)
Supplement: Supplementary file 1 — Supplementary Information. [file 41598_2023_41458_MOESM1_ESM.docx]

**Supplementary material: Llamas (*Llama glama*) enhance proglacial ecosystem development in Cordillera Blanca, Peru**

# **Appendix 1: Method details**

- **Experimental design**

The 8 plots were separated by a minimum of 6 m between each other to avoid strong effects of autocorrelation. We built fences sufficiently large to respect the well-being of the animals without impacting their movement and latrine and grazing behaviors. The fences were made with barbed wire and eucalyptus posts to prevent llamas from escaping the grazing plots, and the 4 corners of the control plots were delimited with wood posts (no wire fence). In addition, we included a buffer zone of 1 m inside the fences to avoid a hypothetical fence effect. The fence wire aperture is about 40 to 50 cm. Therefore, the only animals that could have been unintentionally excluded from the fenced plots are tarucas (*Hippocamelus antisensis*) and vicuñas (*Vicuña vicuña*), however, these herbivores have not been reported within the Pumahuacanca valley. In the absence of the llamas, the doors of the fences were kept open, thus we do not exclude the possibility that other animals could have entered inside the fenced plots. During the three-days llama manipulation, because of the presence of the llamas and farmers in the vicinity of the experiments, it is very unlikely that the plots (llama and/or control) were exposed to herbivory or trampling other than the one caused by the llamas within the fenced plots. The subplots — marked with wood posts buried at the 4 corners —were placed at a minimum distance of 3 m from each other to reduce spatial autocorrelation. A posteriori, we computed species accumulation curves for the 2019 and 2022 data to validate the adequacy of the 64 subplot surveys in representing the vascular richness of the overall experiment.

Considering the difficulty of working with llamas at approximately 4700 m.a.s.l., we carefully considered the animal load and grazing intensity to likely replicate a natural phenomenon. First, we introduced three llamas per enclosure (i.e., llama plots) to respect their well-being (according to the total proglacial area available to build 4 replications of the fenced plot) and nutritional needs, while also ensuring that the animals would not escape joining the rest of the herd within the neighboring plots (e.g., two llamas was too low-density and might have stressed the animals). Second, we applied monthly grazing for 3 consecutive days to limit the visit frequency of the farmers to the experiment from downstream of the valley, thus limiting the anthropogenic impact on the overall experiment. Indeed, during the 3 days of grazing the farmers stay close to the experimental area (approximately 400 m downslope) and do not come back to the village. In addition, to avoid an identity effect of the llamas within the plots, we established a monthly llama rotation between the fenced plots using a calendar set up with the local community. The llamas were tagged with a number to recognize them and distributed them within the grazed plots according to the calendar. During the 3 day stays of the llamas in the plots, the farmers brought the llamas down to the valleys once a day to (1) ensure they had enough forage to complement their diet from the experiment and (2) replicate the natural Andean camelid travel between ranges ^1^. Because of these conditions, we expect the effect of the llamas within the experiment to be like the effect of wild populations. After two months of experimentation, we observed that the presence of the farmers and the llamas at the location of the experiment attracted livestock (cattle and donkeys) from the downstream grazing areas to the experimental zone. Therefore, in August 2019, we built a protection fence approximately 350 m downstream from the experiment to prevent livestock (cattle, sheep, and donkeys) from entering the control plots and damaging the fences of the experiment. The control plots were not subject to trampling effects; therefore, our study investigates the effects of herbivory and trampling.

- **Field data collection**

*Floristic and geomorphic permanent subplot evaluations*

We did not reiterate the floristic subplots evaluation in June 2020 due to the Covid pandemic and our inability to go to the field; instead, we surveyed the experimental plots in December 2020 (Table 1). Vascular plant density was measured as the percentage of foliar tissues within the overall volume of the species. Similarly, the relative necromass was estimated as the percentage of foliar tissues within the overall volume of the species. The average of each species was measured with a ruler as the vertical distance from the ground to the vegetative stem of the plant. We measured slope degree using a Suunto Tandem clinometer. We collected temperature data from the buried data logger once a year from June 2019 to June 2022.

*Leaf sampling*

Because of the shapes and size of the leaf, and their prompt curling after being collected, the measurement of the Specific Leaf Area (SLA) is challenging and may not be the appropriate parameter to reflect the differences in productivity and leaf structure of the different individuals ^2^. Since LDMC, defines leaf construction costs and can be used instead of SLA we favored this trait measurement over SLA. The four species selected for the trait measurement displayed different growth forms (Tussock grass: *Cinnagrostis rigida* and *Agrostis tolucensis*, Shrub: *Senecio sublutescens*, and Subshrub: *Pernettya prostrata*).

Each leaf sample is a composite of a minimum of 3 individuals, summing to 15 to 40 leaves per sample depending on the species: approximately 5 leaves per individual were collected for the gender *Senecio*, *Agrostis,* and *Cinnagrostis* because of their larger sizes, and 10 leaves per individual were collected in the cases of *Pernettya*. We sampled mature leaves from the middle part of the stem of adult individuals and avoided leaves with obvious symptoms of pathogen or herbivore attack. We stored the leaf material in Ziploc bags filled with a humid paper towel and kept them in a cooler until our arrival in the city of Huaraz. We kept the samples refrigerated overnight and transferred them the following morning to the Environmental Quality Laboratory of the Faculty of Environmental Sciences of the Santiago Antúnez de Mayolo National University (UNASAM) in Huaraz for analysis.

*Soil sampling*

For each sample, we collected 300-500 gr of material for laboratory analysis. The samples gathered in June 2022 from the llama plots were sampled downslope or next to dung piles (when possible) at a minimal distance of 1 m from the dung pile. The 2022 samples were air-dried in the city of Huaraz, sieved at 2mm, and sent to the Soil and Geoarchaeology Laboratory of the University of Texas at Austin for further analysis, whereas the samples collected in 2019 were oven dried at 100°C in Huaraz before being sent to our laboratory.

*Feces sampling*

We sampled the feces from the llama dung pile removing the very top layer of the dung to avoid the collection of moss or other external organic material (Figure A1). We sampled in the upslope, mid-slope and downslope portions of the plots to detect a leaching effect from water run-off and therefore explain potential distinct responses of soil composition and plant development to the presence of llamas. For each sample, we collected approximately 800g of material in a Ziploc bag. After homogenization of the sample in the bag, we subsampled approximately half of it for nutrient analysis and a half for the analysis of the presence and viability of seeds. The feces samples for nutrient analysis were stored in the same manner as the leaf material and transferred to the Environmental Quality Laboratory in Huaraz the following morning. The samples for the seeds analysis were transferred the same day to the Herbarium of the Museum of Natural History of the National University of San Marcos in Lima for further analysis.

**Table S1** gives an overview of the datasets collected in the field and measured in the laboratory between 2019 and 2022.


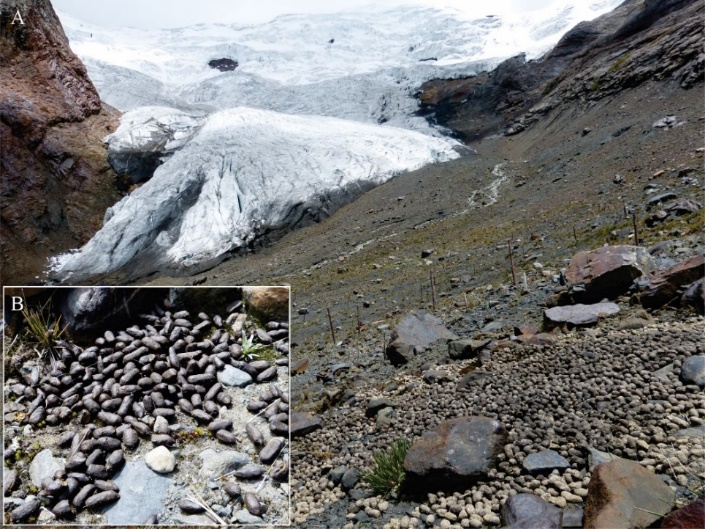


**Figure S1**: Llama dung pile within the llama inclusion experiment at the Uruashraju foreland. (A) Dung pile and (B) llama pellets.

**Table S1:** Characteristics of datasets and dependent and independent variables.

| **Data collected** | **Variable** | **Datatype** | **Unit** | **Support (n)** | **Source** |
| --- | --- | --- | --- | --- | --- |
| Floristic surveys of the permanent subplots | Plant cover | Quantitative, continuous | % | Subplots (64) | Field evaluations |
|  | Vascular plant species | Nominal | - |  |  |
|  | Plant height | Quantitative, continuous | cm |  |  |
|  | Plant necromass | Quantitative, continuous | % |  |  |
|  | Fertility | Binary | - |  |  |
|  | BSC cover | Quantitative, continuous | % |  |  |
|  | Pasture eaten | Binary | - |  |  |
|  | Greenery index | Ordinal | - |  |  |
| Plant functional traits | Leaf Dry Matter Content (LDMC) | Quantitative, continuous | ratio | Plot | Laboratory Analysis |
|  | Leaf Total Nitrogen (PN) | Quantitative, continuous | mg/Kg |  |  |
|  | Leaf Total Phosphorus (PP) | Quantitative, continuous | mg/Kg |  |  |
| Llama dung pile sampling | Seed species | Nominal | - | Llama plot (3x4) | Laboratory analysis |
|  | Number of seed | Quantitative, discrete | - |  |  |
|  | Seed viability | Binary | - |  |  |
| Soil sampling | Soil pH | Quantitative, continuous | - | Plot (3x8) | Soil laboratory analysis |
|  | Clay, Silt, Sand contents | Quantitative, continuous | % |  |  |
|  | Soil organic carbon (SOC) | Quantitative, continuous | % |  |  |
|  | Soil inorganic carbon (IC) | Quantitative, continuous | % |  |  |
|  | Soil total nitrogen (TN) | Quantitative, continuous | % |  |  |
|  | Slope | Quantitative, continuous | % | Subplots (64) | Field evaluations |
| Geomorphic surveys | Sand, Gravel, Rock, Block content | Quantitative, continuous | % |  |  |
|  | Geomorphic activity | Binary | - |  |  |
| Climatic data | Soil temperature | Quantitative, continuous | [deg C] | Plot (2x8) | Field sensors |
|  | Air temperature | Quantitative, continuous | [deg C] | Uruashraju weather station | Autoridad Nacional de Agua |
|  | Precipitation | Quantitative, continuous | [mm] | Uruashraju weather station | Autoridad Nacional de Agua |

- **Laboratory analysis**

*Soil analysis - Loss on Ignition*

We determined soil pH on the dried fine (<2mm) earth samples using a pH probe and a soil: solution ratio of 1:2 using approximately 15g of sample. For soil carbon analysis, first, we estimated SOC, and carbonate mineral content using the LOI method ^3^. The subsamples were first dried at 105°C for 24 hours in a ventilated oven and then ignited at 550°C for 4 hours in a muffle furnace (Cole-Parmer StableTemp Muffle Furnace). We estimated SOC using the conventional equation (SOC (mg/kg) = LOI_550_ (mg/kg) *0.58). We recognize that converting LOI data by multiplication with the conventional conversion factor 0.58 can overestimate the SOC stock ^4^ for soil with high clay content (50 g clay 100g^−1^) or underestimate the SOC stock for soil with a small clay content (10 g 100 g−1). In our case, most samples were sandy clay loam or sandy loam with a clay content between 10 and 30%. Therefore, we consider the conventional conversion factor a good estimate for our work. Finally, the samples were heated at 1000°C for 2 hours to remove carbonates. Calculation of the inorganic carbon (IC) assumes that ignition follows a stoichiometric relationship ^3^, and that the amount of carbonate is 1.36 × LOI_1000_ ^3^. This implies that IC = 0.273 × LOI_1000_. For the Elemental analyzer method, we used a Thermo MAT253 isotope ratio mass spectrometer of the Jackson School of Geosciences, UT Austin, TX, USA, and use soil samples sieved at 250 µm and free of carbonates. Finally, N was measured using an elemental analyzer CHNS-O (ECS 8020 Model, Costech Analytical Technology, INC) at our Soils and Geoarchaeology Laboratory.

*Clay deflocculation and Particle Size Analysis*

Particle Size analysis was on unmilled, sieved (< 2 mm) material (3 to 4.5gr) using a Fritsch Analysette 22 at the Laboratory for Environmental Archaeology of UT Austin. Before particle size analysis, the samples were shaken for 24 h with 0.005% sodium hexametaphosphate to deflocculate the clays. Before adding the material to the sample bath, we used a splitter to help gather the reliable representative samples needed to yield accurate particle size analysis.

*Leaf and feces samples analysis*

Leaf total Nitrogen and Phosphorus were determined respectively using Kjeldahl digestion and Molybdovanadate acid digestion. The fresh leaf samples were weighed upon arrival at the laboratory and then dried for 60min at 105 °C and then weighed again. Dry weight was measured to compute LDMC (ratio of leaf dry mass to leaf saturated mass). The total carbon of the llama feces was measured using disintegration with sulfuric acid and peroxodisulfate and photometric method. Since we collected our samples only within the dung piles, we consider that the total carbon concentration measured corresponds to the Total Organic Carbon (TOC) concentration within the dung piles, and therefore is comparable to our measures of SOC on the soil samples.

*Seed viability*

For the analysis of presence and viability of seeds, first the samples were oven-dried at 23 C° for 3 days ^5,6^. We analyzed only fecal pellets to be sure to study seeds ingested by llamas. The final dung pile pellet samples analyzed weighed between 6.5 and 15g. We identified the seed species with botanical specialist support and using a list of the plant species present in the studied area (**Table S3: Supplementary material**), and seed reference collections (Seed Bank of the Plant Physiology Laboratory of the UNMSM). Then, we apply the Tetrazolium test to test the viability of the seed following França-Neto & Krzyzanowski ^7^. Part of the pericarp was removed from all seeds and submerged in distilled water for 24 hours and later for 24 hours in a 1% 2,3,5-triphenyl-2H-tetrazolium solution. After this treatment, seeds were then classified as (1) viable, when embryos were totally dyed, and (2) not viable, when embryos were partially dyed or not dyed.

- **Climatic data collection and analysis**

The 16 data loggers registered soil temperature data every 4 hours from 0. However, due to the Covid pandemic and our inability to go to the field in June 2020 to replace their batteries, some of the sensors stopped registering temperature and several data gaps were present between 22.05.2021 and 09.06.2022. Therefore, for our analysis we used the period from 23.06.2019 to 22.05.2021. Since the upper logger of the control plot number 4 was lost between May 2019 and June 2021 resulting in incomplete data for the period studied, we computed the same Wilcoxon test including only 3 of the 4 control plots. We found the same result and therefore did not report them.

The complementary climatic data provided by the ANA (Autoridad National del Agua, Huaraz, Peru) was registered at the Uruashraju glacier foreland weather station (Figure S1). To infer the role of variations in air temperatures on our response variables we used the air temperature data collected between June 2018 and May 2022. We divided the data in three periods corresponding to the temperature preceding our dry season field evaluation (May 2019, May 2021, and June 2022): June 2018 to May 2019; June 2019 to May 2021 and June 2021 to May 2022. We did not include a May 2019-December 2020 or December 2020 to May 2021 periods since the results were going to be biased because of the different seasonality (i.e., different number of warm and cold months). The existing precipitation data was complete only for the period from October 2017 to October 2020, therefore we could not infer any changes in precipitation between 2021 and 2022 and we reported mean daily precipitation per month for the 2017 to 2022 period.

# **Appendix 2: Supplementary figures and tables**


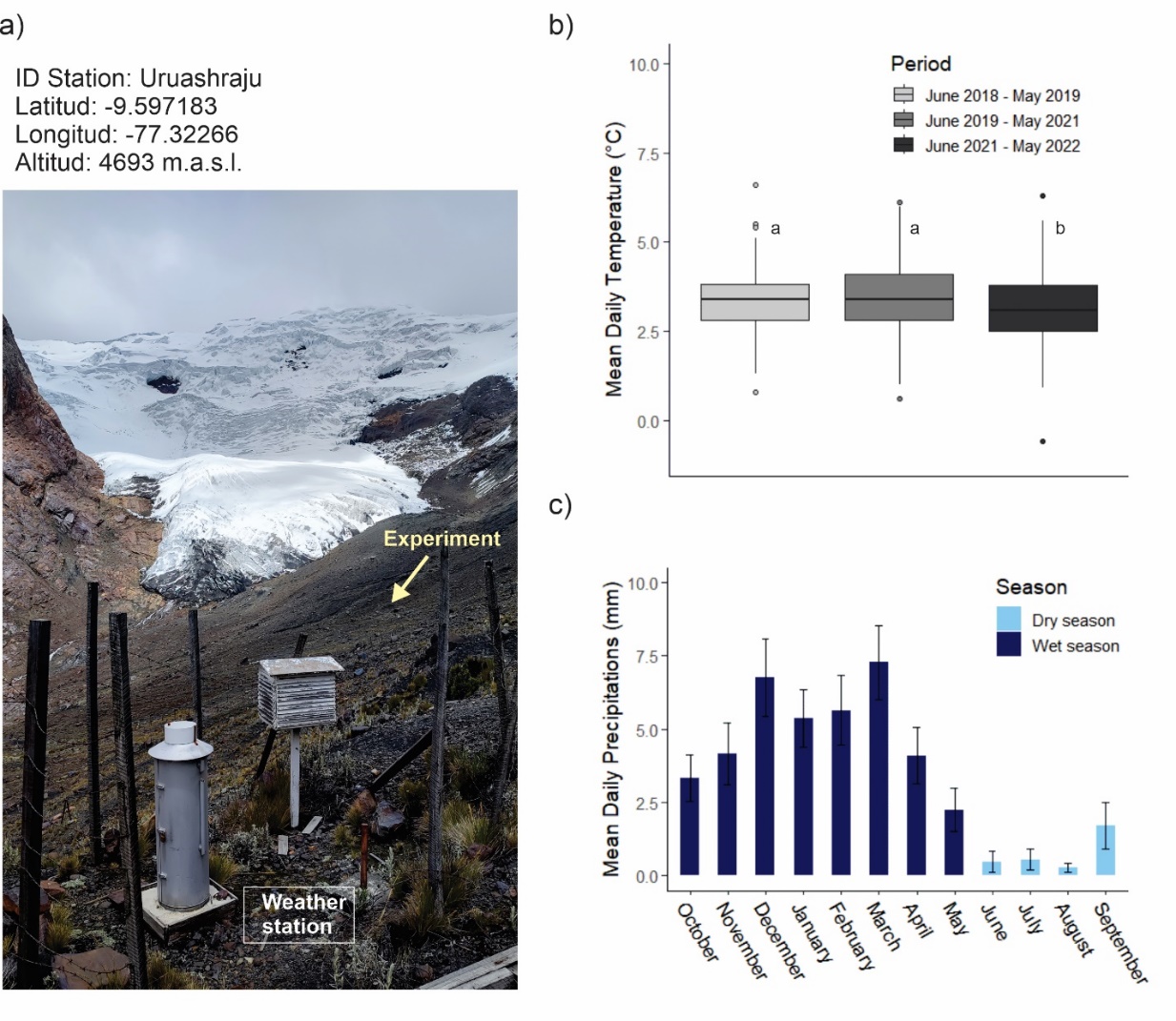


**Figure S2:** Climatic data at the Uruahsraju weather station. (a) Location and coordinates of the weather station with respect to the experiment. (b) Mean daily air temperature (°Celsius) from June 2018 to May 2022. Letters represent the significantly different groups according to Tukey Honestly Significant Difference tests (p≤0.05). Results of the Tukey HSD test are presented in **Table S2**. (c) Mean daily precipitations (mm) per month between October 2017 and September 2020. Data was provided by the ANA (Autoridad Nacional del Agua, Huaraz, Peru).

**Table S2:** Tukey's ‘Honestly Significant Difference confidence intervals and significances for the Air Temperature data between June 2018 and May 2022. Period 1 is from June 2018 to May 2019. Period 2 is from June 2019 to May 2021, and Period 3 is from June 2021 to May 2022. P-values in bold are significant at 0.05.

| comparison | diff | lwr | upr | p adj |
| --- | --- | --- | --- | --- |
| Period2-Period1 | 0.083699 | -0.09352 | 0.260922 | 0.509168 |
| Period3-Period1 | -0.22241 | -0.41696 | -0.02786 | **0.02023** |
| Period3-Period2 | -0.30611 | -0.44501 | -0.16721 | **8.07E-07** |

**Figure S3.** Soil particle size distribution – the percentage of clay, silt, and sand – of the controls (dark magenta points) and llama (dark orange points) soil samples collected in (A) 2019 (triangles) and (B) 2022 (full circles). The abbreviated names of the USDA texture class polygons correspond to sand (Sa), loamy sand (LoSa), sandy loam (SaLo), loam (Lo), sandy clay loam (SaClLo), sandy clay (SaCl), clay loam (ClLo), silty loam (SiLo), silty clay loam (SiClLo), silty clay (SiCl), silt (Si) and clay (Cl).


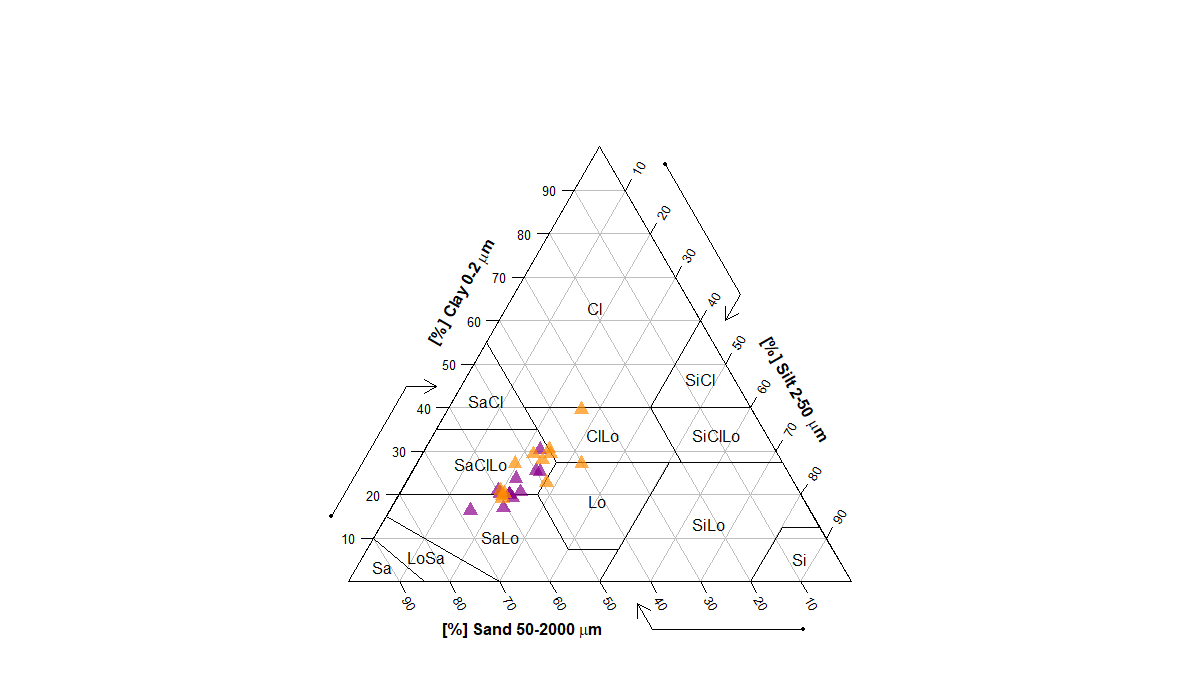

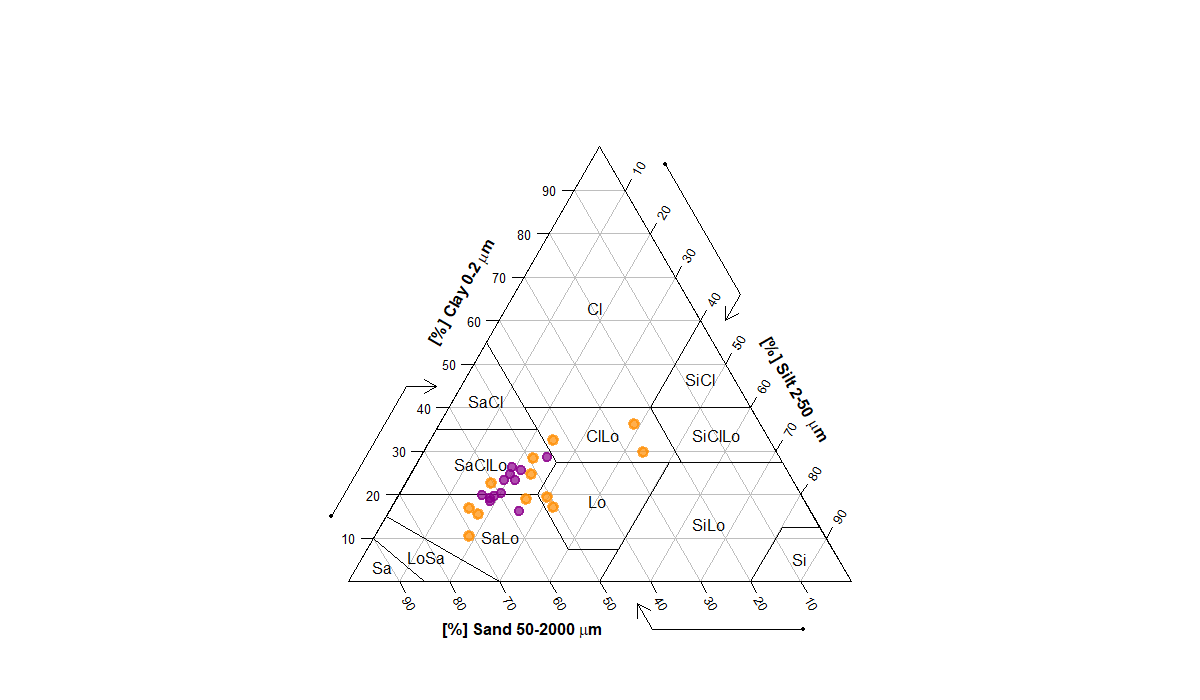


A

B

**Table S3** – List of species found at the Uruashraju glacier foreland (from the position of the glacier forefront in 2019 to the Little Ice Age glacial extension (LIA); Zimmer et al., unpublished). Distance from glacier is the approximate distance from the glacier tongue in 2019 calculated in ArcGISPro and based on the glacier outline provided by the ANA (Autoridad Nacional del Agua, Peru).

| **Species** | **Age groups:** | **0-14 yrs** | **14-31yrs** | **31-57yrs** | **57-71yrs** | **LIA** |
| --- | --- | --- | --- | --- | --- | --- |
| Distance from glacier: | | 0**-**200 m | 200**-**500m | 500**-**770m | 770**-**930m | ∼2.2 km |
| *Achyrocline alata* |  |  |  |  | x | x |
| *Agrostis tolucensis* |  | x | x | x | x |  |
| *Alchemilla pinnata* |  |  |  |  | x | x |
| *Baccharis caespitosa* |  |  |  |  | x | x |
| *Bartsia diffusa* |  |  |  |  | x |  |
| *Belloa piptolepis* |  |  |  |  | x |  |
| *Cinnagrostis brevifolia* |  |  |  |  |  | x |
| *Cinnagrostis coronalis* |  | x | x | x | x |  |
| *Cinnagrostis ovata* |  | x |  | x |  |  |
| *Cinnagrostis recta* |  |  |  |  |  | x |
| *Cinnagrostis rigida* |  | x | x | x | x |  |
| *Cinnagrostis vicunarum* |  |  |  |  |  | x |
| *Chaerophyllum andicola* |  |  |  |  |  | x |
| *Chersodoma ovopedata* |  |  |  | x | x |  |
| *Distichia muscoides* |  |  |  |  |  | x |
| *Elaphoglossum sp.* |  |  |  |  |  | x |
| *Epilobium nivale* |  |  |  |  |  | x |
| *Gentiana thyrsoidea* |  |  |  |  |  | x |
| *Gentianella thyrsoidea* |  |  |  |  | x |  |
| *Hieracium sp* |  |  |  |  |  | x |
| *Lobelia oligophylla* |  |  |  |  |  | x |
| *Loricaria ferruginea* |  |  |  | x | x |  |
| *Lucilia conoidea* |  | x |  | x | x |  |
| *Lucilia kunthiana* |  |  |  | x | x | x |
| *Lupinus aff. microphyllus* |  |  |  |  |  | x |
| *Lupinus sp.* |  |  |  |  |  | x |
| *Luzula racemosa* |  |  |  |  | x | x |
| *Melpomene peruviana* |  |  | x | x | x | x |
| *Mniodes longifolia* |  |  |  |  | x | x |
| *Muehlenbeckia volcanica* |  |  |  | x | x | x |
| *Muhlenbergia peruviana* |  |  |  |  |  | x |
| *Oritrophium limnophilum* |  |  |  |  |  | x |
| *Pernettya prostrata* |  | x | x | x | x | x |
| *Phlegmariurus crassus* |  |  |  | x | x |  |
| *Plantago lamprophylla* |  |  |  |  |  | x |
| *Plantago tubulosa* |  |  |  |  |  | x |
| *Polystichum orbiculatum* |  |  | x |  | x |  |
| *Ribes cuneifolium* |  |  |  |  |  | x |
| *Senecio macrorrhizus* |  |  |  |  | x | x |
| *Senecio rufescens* |  |  |  |  | x |  |
| *Senecio sublutescens* |  | x | x | x | x | x |
| *Senecio sulinicus* |  |  |  |  | x |  |
| *Trichophorum rigidum* |  |  |  |  |  | x |
| *Werneria nubigena* |  |  |  | x |  | x |
| *Werneria pygmaea* |  |  |  |  |  | x |
| *Werneria villosa* |  |  |  |  |  | x |

**Table S4** – List of species found within the experiment, Control and Llamas mean presence or absence within the plots for the year presented. Total cover corresponds to the total cover of each species over the overall experiment. Dispersal Mode: anemochory (A), endozoochory (EZ). Cover values are in percent (%).

| **Nombre** | **Autor** | **Family** | **Growth form** | **Dispersal mode** | **Habitat** | **Control 2019** | **Control 2022** | **Llamas 2019** | **Llamas 2022** | **Total cover** | **Cover llama plots** | **Palatability for Andean camelid** |
| --- | --- | --- | --- | --- | --- | --- | --- | --- | --- | --- | --- | --- |
| *Agrostis tolucensis* | Kunth | Poaceae | Tussock grass | A | Disturbed areas, grasslands | yes | yes | yes | yes | 4.43 | 2.58 | Yes ^9^ |
| *Belloa piptolepis* | (Wedd.) Cabrera | Asteraceae | Herb | A | Grasslands, shrublands | no | no | no | yes | 0.05 | 0.11 |  |
| *Cinnagrostis ovata* |  | Poaceae | Tussock grass | A | Grasslands, riversides, swamps | yes | yes | yes | no | 0.5 | 0.24 | Yes - field obs. |
| *Cinnagrostis rigida* | (Kunth) P.M. Peterson, Soreng, Romasch. & Barberá | Poaceae | Tussock grass | A | Grassland | yes | yes | yes | yes | 50.6 | 45.2 | Yes - field ob. |
| *Cinnagrostis vicunarum* | (Wedd.) P.M. Peterson, Soreng, Romasch. & Barberá | Poaceae | Tussock grass | A | Grasslands, rocky slopes | no | yes | no | yes | 0.12 | 0.04 | Yes ^9,10^ |
| *Gnaphalium polium* | Wedd. | Asteraceae | Rosette | A | Grasslands, shrublands | no | no | yes | yes | 0.46 | 0.97 |  |
| *Luzula racemosa* | Desv. | Cyperaceae | Tussock grass | A | Grasslands, rocky slopes | no | yes | no | yes | 0.03 | 0.04 | No ^11^ |
| *Melpomene peruviana* | (Desv.) A.R. Sm. & R.C. Moran | Polypodiaceae | Herb | - | Zone protected by | yes | yes | no | no | 0.12 | 0 |  |
|  |  |  |  |  | boulders |  |  |  |  |  |  |  |
| *Mniodes kunthiana* | (DC.) S.E. Freire, Chemisquy, Anderb. & Urtubey | Asteraceae | Rosette | A | Grasslands, shrublands | yes | yes | yes | yes | 18.3 | 12.9 |  |
| *Oritrophium limnophilum* | (Sch. Bip.) Cuatrec. | Asteraceae | Acaulescent rosette | A | n.d | no | no | no | yes | 0.11 | 0.24 |  |
| *Pernettya prostrata* | (Cav.) DC. | Ericaceae | Subshrub | EZ | Disturbed areas, forests, grasslands, rocky slopes | yes | yes | yes | yes | 2.51 | 1.36 | Yes - field obs. |
| *Senecio rufescens* | DC. | Asteraceae | Erect shrub | A | Rocky subnival puna | no | no | no | yes | 0.05 | 0.1 | Yes - field obs. |
| *Senecio sublutescens* | Cuatrec. | Asteraceae | Herb | A | Rocky subnival puna | yes | yes | yes | yes | 22.7 | 36.2 |  |
| *Sp.* |  | Asteraceae | Herb |  |  | no | no | no | yes | 0.03 | 0.06 |  |

**Table S5.** Summary statistics for the environmental characteristic of the llama and control plots. The daily temperature minimum and maximum correspond respectively to the minimum of the daily minimum and the maximum of the daily maximum.

|  | **Control** | **Llama** | **p.value** | **p.signif** |
| --- | --- | --- | --- | --- |
| **Slope** | 23.81 (11.51) | 26.03 (12.96) | 0.44 | ns |
| **Granulometry** |  |  |  |  |
| Sand | 18.91 (18.15) | 31.89 (28.74) | 0.078 | ns |
| Gravel | 16.09 (11.57) | 15.52 (13.67) | 0.59 | ns |
| Rock | 35.71 (20.23) | 31.13 (22.15) | 0.37 | ns |
| Block | 10.14 (11.35) | 6.41 (7.57) | 0.3 | ns |
| **Daily temp** |  |  |  |  |
| mean | 7.65 (2.09) | 7.67 (2.21) | 0.96 | ns |
| min | 0.01 (1.28) | -0.44 (1.23) | 3.80E-10 | *** |
| max | 29.55 (4.79) | 33.43 (5.9) | 0.001 | *** |

**Table S6.** Summary statistics for Clay, Silt and Sand contents, pH, IC by LOI, SOC by LOI and EA methos, and δ13C in control and llama plots, in 2019 and 2022.

|  | **2019** | | **2022** | |  |  | **2019** | | **2022** | |
| --- | --- | --- | --- | --- | --- | --- | --- | --- | --- | --- |
|  | *Control* | *Llama* | *Control* | *Llama* |  |  | *Control* | *Llama* | *Control* | *Llama* |
| **Clay (%)** |  |  |  |  |  | **SOC by LOI (%)** | |  |  |  |
| Max | 30.39 | 39.54 | 28.56 | 36.09 |  | Max | 2.08 | 2.16 | 2.12 | 2.67 |
| Mean | 21.67 | 26.17 | 22.12 | 22.72 |  | Mean | 1.61 | 1.59 | 1.74 | 1.94 |
| Min | 16.41 | 18.95 | 16.16 | 10.5 |  | Min | 1.16 | 1.11 | 1.34 | 1.55 |
| SD | 3.97 | 5.95 | 3.72 | 7.68 |  | SD | 0.26 | 0.3 | 0.24 | 0.32 |
| **Silt (%)** |  |  |  |  |  |  |  |  |  |  |
| Max | 25.38 | 32.81 | 25.91 | 43.82 |  | **SOC by EA (%)** | |  |  |  |
| Mean | 21.86 | 23.81 | 20.52 | 25.93 |  | Max |  |  | 1.62 | 1.67 |
| Min | 16.06 | 19.5 | 16.73 | 15.47 |  | Mean |  |  | 1.22 | 1.38 |
| SD | 2.52 | 3.97 | 2.66 | 8.83 |  | Min |  |  | 0.93 | 0.98 |
| **Sand (%)** |  |  |  |  |  | SD |  |  | 0.17 | 0.22 |
| Max | 67.51 | 59.95 | 63.37 | 70.67 |  |  |  |  |  |  |
| Mean | 56.45 | 50.01 | 57.34 | 51.34 |  |  |  |  |  |  |
| Min | 46.72 | 33.93 | 46.24 | 25.1 |  | **IC by LOI (%)** | |  |  |  |
| SD | 5.72 | 8.42 | 4.93 | 14.59 |  | Max | 0.98 | 1.02 | 1 | 1.26 |
| **pH** |  |  |  |  |  | Mean | 0.76 | 0.75 | 0.82 | 0.92 |
| Max | 3.96 | 3.9 | 4.35 | 5.34 |  | Min | 0.55 | 0.52 | 0.63 | 0.73 |
| Mean | 3.61 | 3.7 | 4.06 | 4.31 |  | SD | 0.12 | 0.14 | 0.11 | 0.15 |
| Min | 3.19 | 3.58 | 3.6 | 3.38 |  |  |  |  |  |  |
| SD | 0.23 | 0.1 | 0.2 | 0.55 |  |  |  |  |  |  |
| **N (%)** |  |  |  |  |  | **δ13C (‰)** |  |  |  |  |
| Max | 0.13 | 0.12 | 0.19 | 0.22 |  | Max |  |  | -24.4 | -24.9 |
| Mean | 0.09 | 0.08 | 0.14 | 0.19 |  | Mean |  |  | -25.09 | -25.32 |
| Min | 0.06 | 0.06 | 0.08 | 0.16 |  | Min |  |  | -25.8 | -26 |
| SD | 0.02 | 0.02 | 0.04 | 0.02 |  | SD |  |  | 0.36 | 0.4 |

**Table S7.** Full linear models for clay, silt, and sand contents, comparing the control and llama plots and including the 2019 baseline and 2022 data. Bold values indicate significance (p≤0.05).


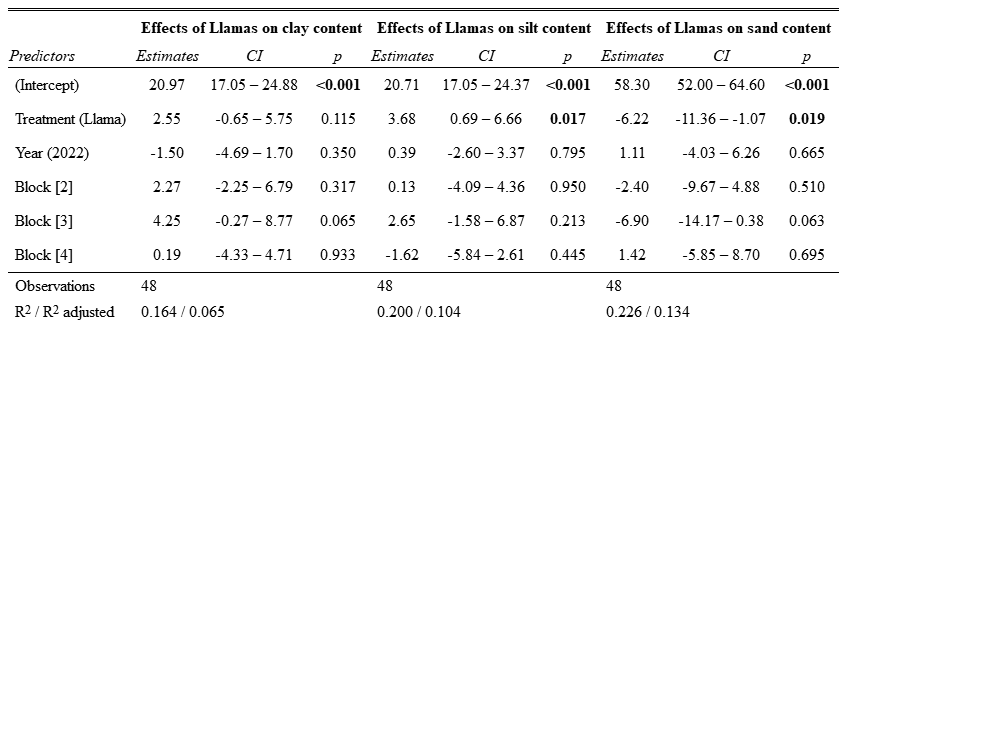


**Table S8:** Coefficients of the 2019 and 2022 linear models for clay, silt, and sand contents. Bold values indicate significance (p≤0.05).


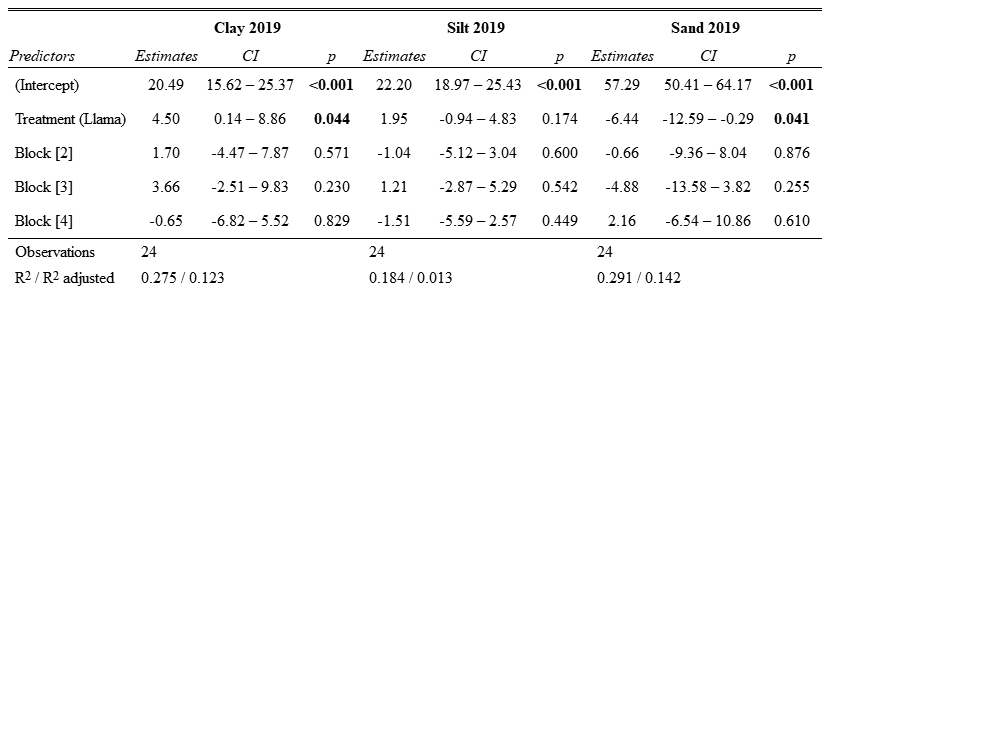


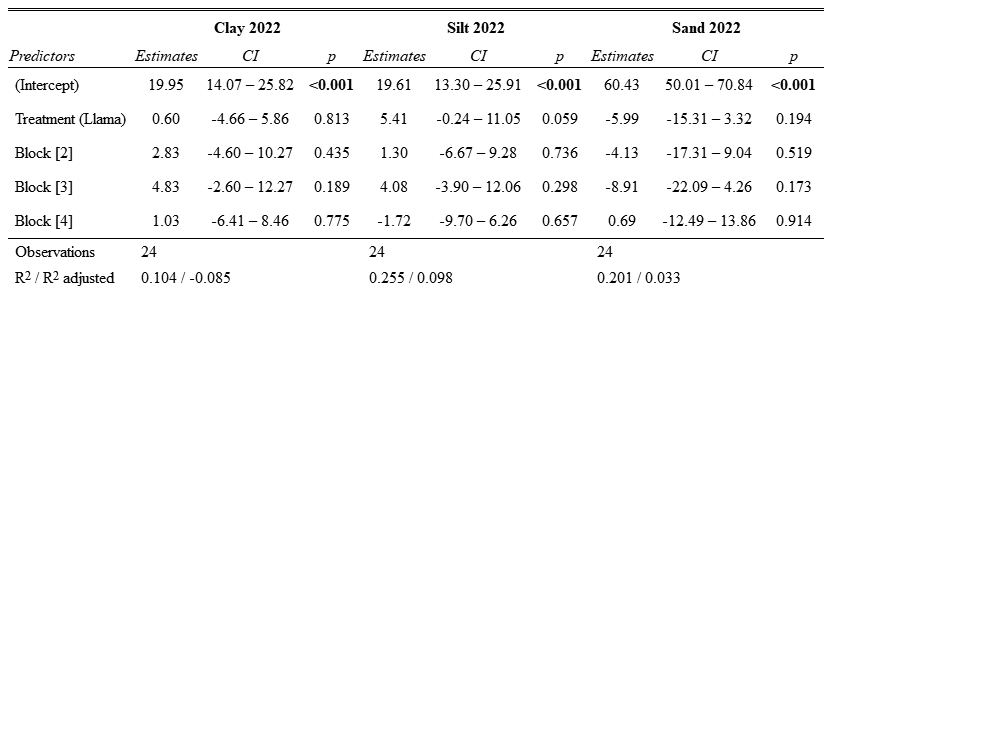


**Table S9:** Coefficients of the 2019 and 2022 linear models for soil organic carbon (SOC, by LOI and EA), δ13C, pH, soil inorganic carbon (IC, by LOI), and total nitrogen (N, by EA). Bold values indicate significance (p≤0.05).


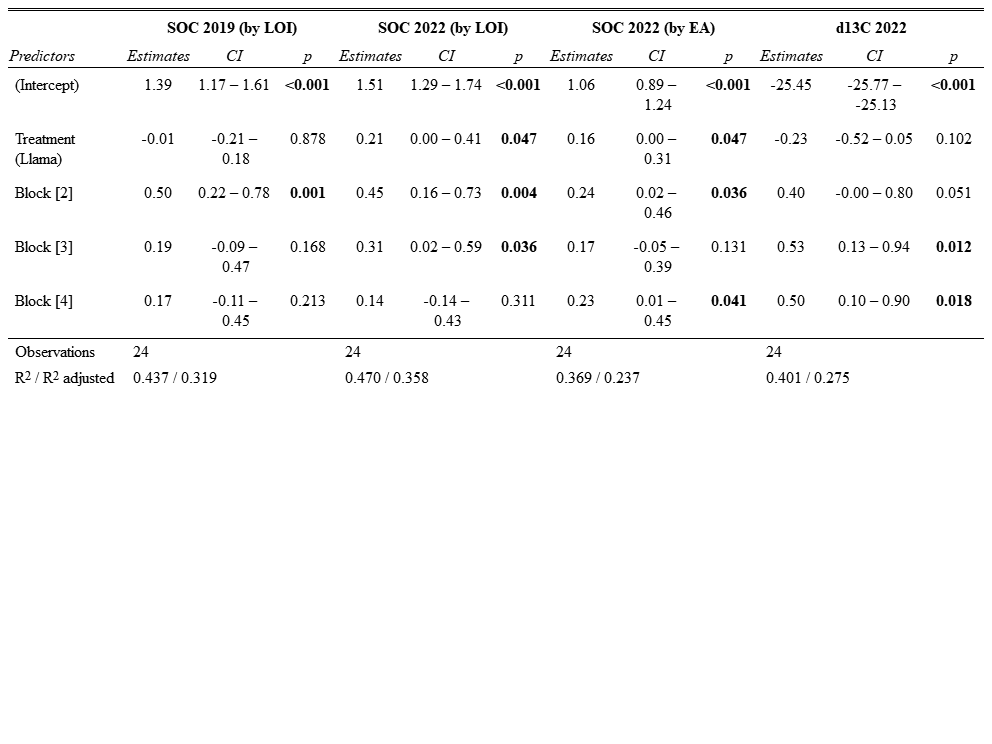


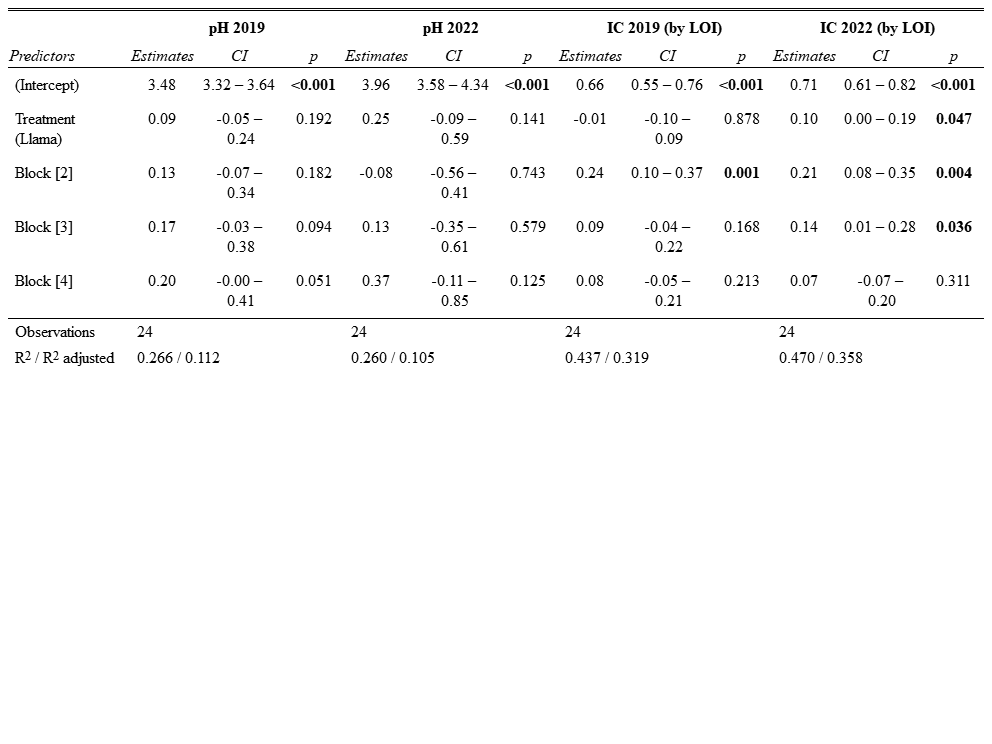


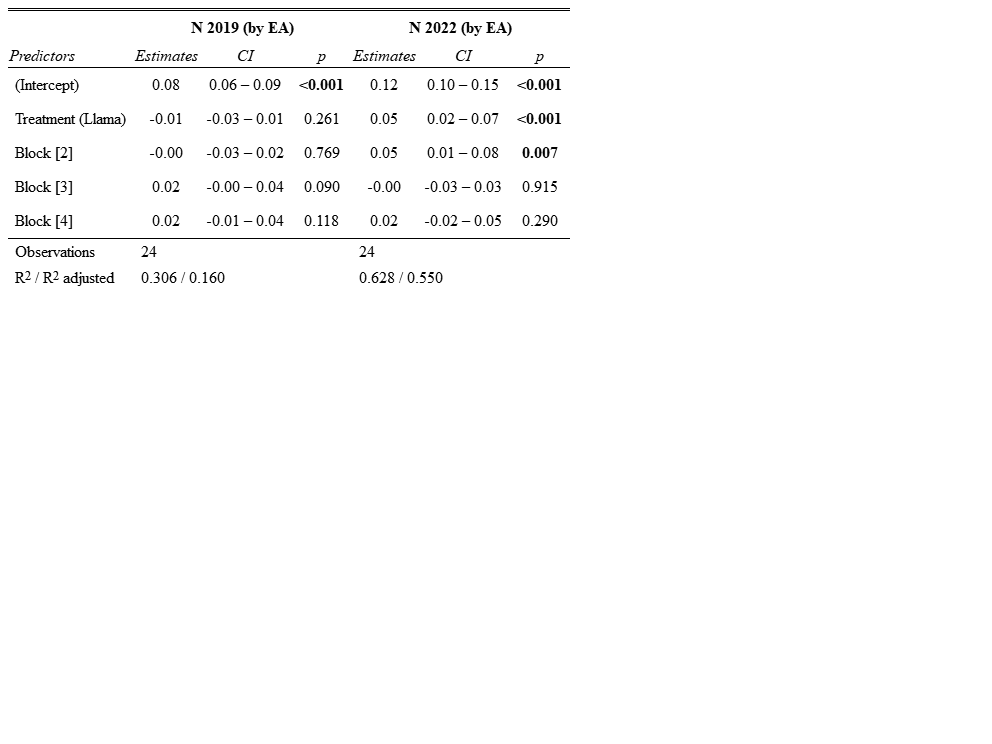


**Table S10.** Full linear models for soil pH, soil organic carbon (SOC, by LOI method), and soil inorganic carbon (IC, by LOI method) contents, comparing the control and llama plots and including the 2019 baseline and 2022 data. Bold values indicate significance (p≤0.05).


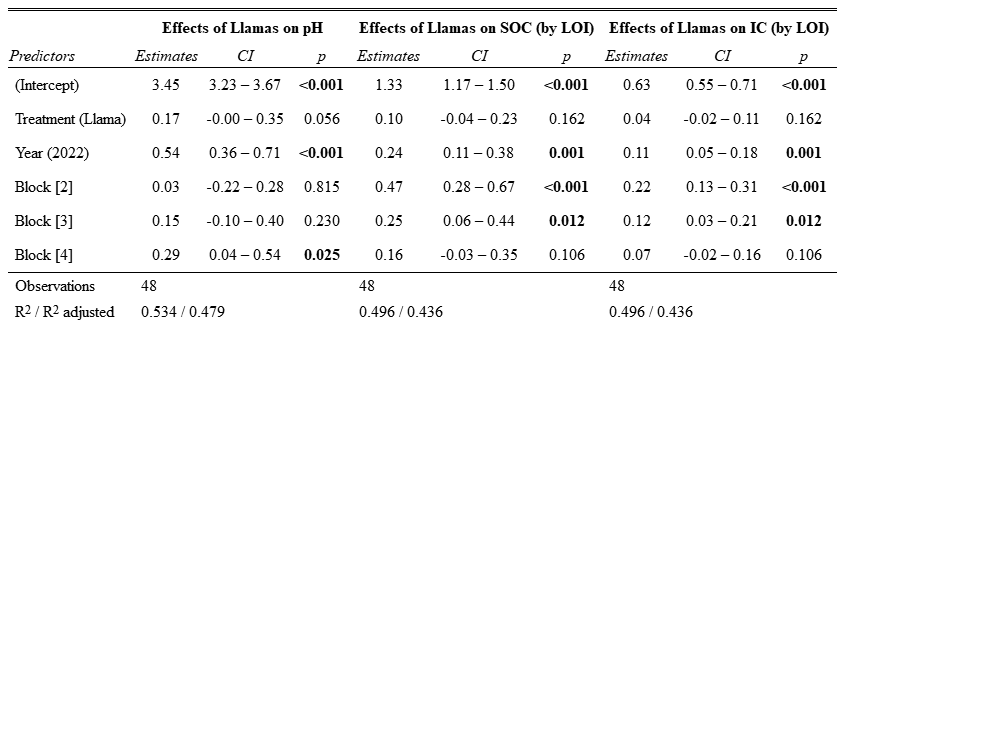


**Table S11:** Composition in total organic carbon (TOC), total nitrogen (TN) and total phosphorus (P) of the llama dung piles. n=12.

| Parameter | Mean | sd |
| --- | --- | --- |
| TOC (%) | 15.4 | 2.99 |
| TN (%) | 5.07 | 1.34 |
| P (%) | 0.12 | 0.01 |

**Table S12.** Summary statistics for vascular plant cover (a.), richness, greenness, height, necromass and fertility (b.), in control and llama plots, in 2019, 2020, 2021 and 2022.

| a. | **Cover** | | | | | | | |
| --- | --- | --- | --- | --- | --- | --- | --- | --- |
|  | **Control** | **Llama** | **Difference** | **Percent difference** | **Fixed effect: Llama treatment** | | | |
|  |  |  |  |  | Estimate | Statistic | p.value | p.signif |
| **June 2019** |  |  |  |  |  |  |  |  |
| mean | 11.38 | 7.96 | -3.42 | -17.69 | -0.31 | -1.58 | 0.12 | NA |
| sd | 14.27 | 9.33 |  |  |  |  |  |  |
| max | 72.50 | 45.00 | -27.50 | -23.40 |  |  |  |  |
| min | 0.50 | 0.00 | -0.50 | -100.00 |  |  |  |  |
| **December 2020** |  |  |  |  |  |  |  |  |
| mean | 8.89 | 8.27 | -0.63 | -3.64 | 0.13 | 2.19 | **0.03** | ***** |
| sd | 8.91 | 10.21 |  |  |  |  |  |  |
| max | 40.50 | 54.00 | 13.50 | 14.29 |  |  |  |  |
| min | 0.50 | 0.00 | -0.50 | -100.00 |  |  |  |  |
| **May 2021** |  |  |  |  |  |  |  |  |
| mean | 9.63 | 8.91 | -0.71 | -3.84 | 0.10 | 1.45 | 0.15 | NA |
| sd | 9.35 | 9.88 |  |  |  |  |  |  |
| max | 42.20 | 47.00 | 4.80 | 5.38 |  |  |  |  |
| min | 0.30 | 0.00 | -0.30 | -100.00 |  |  |  |  |
| **June 2022** |  |  |  |  |  |  |  |  |
| mean | 12.64 | 13.97 | 1.32 | 4.97 | 0.33 | 3.94 | **0.0002** | ******* |
| sd | 13.22 | 14.11 |  |  |  |  |  |  |
| max | 59.00 | 62.00 | 3.00 | 2.48 |  |  |  |  |
| min | 0.70 | 0.00 | -0.70 | -100.00 |  |  |  |  |

| b. | |  | **Richness** | | | | |  | **Greeness** | | | | |  | **Height** | | | | |  | **Necromass** | | | | |  | **Fertility** | | |
| --- | --- | --- | --- | --- | --- | --- | --- | --- | --- | --- | --- | --- | --- | --- | --- | --- | --- | --- | --- | --- | --- | --- | --- | --- | --- | --- | --- | --- | --- |
|  | |  | **Control** | | | **Llama** | |  | **Control** | | | **Llama** | |  | **Control** | | | **Llama** | |  | **Control** | | | **Llama** | |  | **Control** | | **Llama** |
|  | |  |  |  |  |  |  |  |  |  |  |  |  |  |  |  |  |  |  |  |  |  |  |  |  |  |  |  |  |
| **June 2019** | |  |  | | |  | |  |  | | |  | |  |  | | |  | |  |  | | |  | |  |  | |  |
| mean | |  | 2.47 | | | 2.19 | |  | na | | | na | |  | 16.18 | | | 18.68 | |  | 1.06 | | | 0.91 | |  | 52.76 | | 57.86 |
| sd | |  | 0.98 | | | 1.09 | |  | na | | | na | |  | 9.11 | | | 10.27 | |  | 1.05 | | | 0.86 | |  | 33.73 | | 35.16 |
| max | |  | 5 | | | 5 | |  | na | | | na | |  | 34.38 | | | 42.83 | |  | 4 | | | 3.81 | |  | 100 | | 100 |
| min | |  | 1 | | | 0 | |  | na | | | na | |  | 1 | | | 4 | |  | 0 | | | 0 | |  | 0 | | 0 |
| **December 2020** |  | | |  |  | |  | | |  |  | |  | | |  |  | |  | | |  |  | |  | | |  |  |
| mean | |  | 2.53 | | | 2.31 | |  | 0.75 | | | 1.16 | |  | 10.88 | | | 12.3 | |  | 8.68 | | | 14.34 | |  | 0 | | 2.19 |
| sd | |  | 0.95 | | | 1.18 | |  | 0.72 | | | 0.88 | |  | 7.62 | | | 7.22 | |  | 10.59 | | | 12.8 | |  | 0 | | 9.41 |
| max | |  | 5 | | | 5 | |  | 2 | | | 2 | |  | 33.5 | | | 39.81 | |  | 37.27 | | | 40.74 | |  | 0 | | 50 |
| min | |  | 1 | | | 0 | |  | 0 | | | 0 | |  | 1 | | | 3.67 | |  | 0 | | | 0 | |  | 0 | | 0 |
| **May 2021** | |  |  | | |  | |  |  | | |  | |  |  | | |  | |  |  | | |  | |  |  | |  |
| mean | |  | 2.75 | | | 2.50 | |  | 0.06 | | | 0.31 | |  | 11.42 | | | 11.27 | |  | 22.44 | | | 27.07 | |  | 22.76 | | 11.3 |
| sd | |  | 1.05 | | | 1.30 | |  | 0.25 | | | 0.69 | |  | 6 | | | 7.42 | |  | 13.61 | | | 16.08 | |  | 32.31 | | 21.37 |
| max | |  | 5 | | | 6 | |  | 1 | | | 2 | |  | 28.17 | | | 34.87 | |  | 50 | | | 66 | |  | 100 | | 75 |
| min | |  | 1 | | | 0 | |  | 0 | | | 0 | |  | 1 | | | 2.07 | |  | 0 | | | 3.78 | |  | 0 | | 0 |
| **June 2022** | |  |  | | |  | |  |  | | |  | |  |  | | |  | |  |  | | |  | |  |  | |  |
| mean | |  | 2.94 | | | 3.25 | |  | 0.06 | | | 0.63 | |  | 11.28 | | | 11.92 | |  | 23.54 | | | 17.47 | |  | 64.9 | | 39.17 |
| sd | |  | 0.91 | | | 1.44 | |  | 0.25 | | | 0.83 | |  | 6.17 | | | 6.9 | |  | 12.82 | | | 7.84 | |  | 31.58 | | 35.63 |
| max | |  | 5 | | | 6 | |  | 1 | | | 2 | |  | 26.67 | | | 27.76 | |  | 57.63 | | | 32.62 | |  | 100 | | 100 |
| min | |  | 2 | | | 0 | |  | 0 | | | 0 | |  | 1.07 | | | 2.42 | |  | 3.62 | | | 0 | |  | 0 | | 0 |

**Table S13.** Regression models for Plant Cover, Plant Richness and Greenness, comparing the control and llama plots.


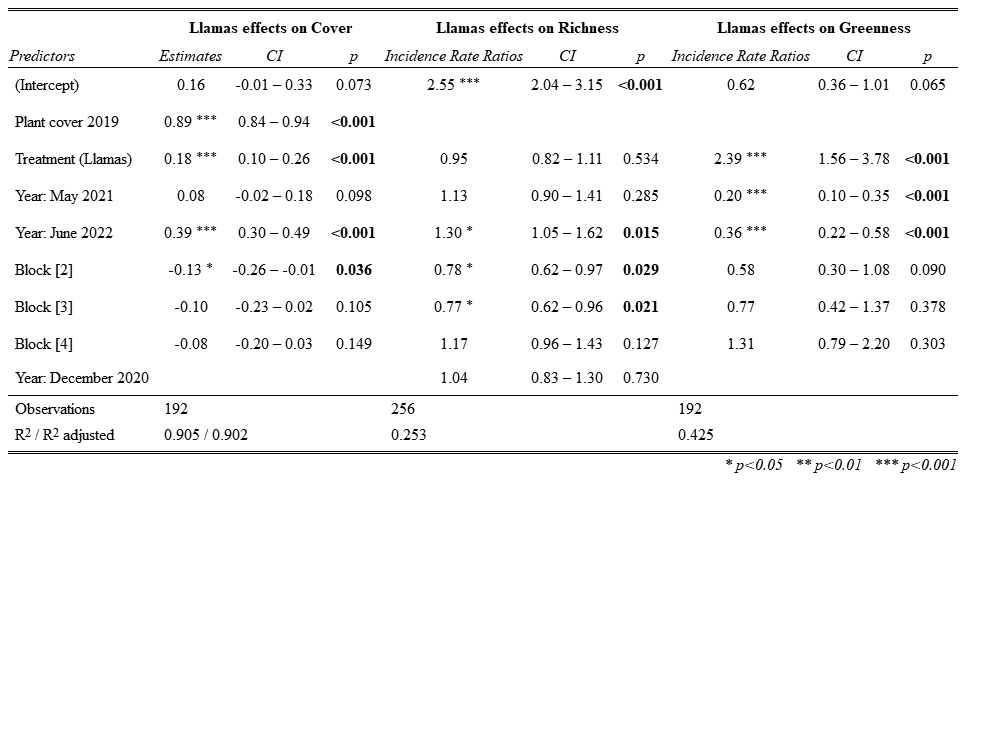


**Table S14** – Results of estimated marginal means and contrast tests for multiple comparisons for the plant cover. p-values adjusted with the Bonferroni procedure. The estimated marginal means and confidence intervals were back transformed (a). Contrast tests were performed on the log scale (b) (not back-transformed). p-values adjusted with the Bonferroni procedure.

| (a) |  |  |  |  |  |  |
| --- | --- | --- | --- | --- | --- | --- |
| **Treatment** | **Years** | **response** | **SE** | **df** | **lower.CL** | **upper.CL** |
| Control | December 2020 | 7.876 | 0.366 | 184 | 7.183 | 8.628 |
| Llama | December 2020 | 9.631 | 0.466 | 184 | 8.750 | 10.591 |
| Control | May 2021 | 8.642 | 0.397 | 184 | 7.889 | 9.458 |
| Llama | May 2021 | 10.548 | 0.506 | 184 | 9.591 | 11.591 |
| Control | June 2022 | 12.174 | 0.543 | 184 | 11.145 | 13.289 |
| Llama | June 2022 | 14.778 | 0.692 | 184 | 13.470 | 16.203 |

| (b) |  |  |  |  |  |
| --- | --- | --- | --- | --- | --- |
| **contrast** | **estimate** | **SE** | **df** | **t.ratio** | **p.value** |
| Control December 2020 - Llama December 2020 | -0.180 | 0.042 | 184.000 | -4.330 | ≤0.001 |
| Control December 2020 - Control May 2021 | -0.083 | 0.050 | 184.000 | -1.665 | 1 |
| Control December 2020 - Control June 2022 | -0.395 | 0.050 | 184.000 | -7.945 | ≤0.001 |
| Llama December 2020 - Llama May 2021 | -0.083 | 0.050 | 184.000 | -1.665 | 1 |
| Llama December 2020 - Llama June 2022 | -0.395 | 0.050 | 184.000 | -7.945 | ≤0.001 |
| Control May 2021 - Llama May 2021 | -0.180 | 0.042 | 184.000 | -4.330 | ≤0.001 |
| Control May 2021 - Control June 2022 | -0.312 | 0.050 | 184.000 | -6.280 | ≤0.001 |
| Llama May 2021 - Llama June 2022 | -0.312 | 0.050 | 184.000 | -6.280 | ≤0.001 |
| Control June 2022 - Llama June 2022 | -0.180 | 0.042 | 184.000 | -4.330 | ≤0.001 |

**Table S15** – Results of the emmeans and contrast tests for multiple comparisons for the subplot greenness. Intervals are back-transformed from the log scale (a). Contrast tests were performed on the log scale (b). p-values adjusted with the Bonferroni procedure.

| (a) |  |  |  |  |  |
| --- | --- | --- | --- | --- | --- |
| **Treatment** | **Years** | **rate** | **SE** | **asymp.LCL** | **asymp.UCL** |
| Control | December 2020 | 0.537 | 0.111 | 0.358 | 0.804 |
| Llama | December 2020 | 1.285 | 0.190 | 0.962 | 1.716 |
| Control | May 2021 | 0.106 | 0.035 | 0.055 | 0.202 |
| Llama | May 2021 | 0.253 | 0.075 | 0.141 | 0.453 |
| Control | June 2022 | 0.194 | 0.052 | 0.115 | 0.327 |
| Llama | June 2022 | 0.463 | 0.104 | 0.298 | 0.721 |

| (b) |  |  |  |  |
| --- | --- | --- | --- | --- |
| **contrast** | **ratio** | **SE** | **z.ratio** | **p.value** |
| Control December 2020 / Llama December 2020 | 0.418 | 0.094 | -3.877 | 0.002 |
| Control December 2020 / Control May 2021 | 5.083 | 1.605 | 5.149 | ≤0.001 |
| Control December 2020 / Control June 2022 | 2.773 | 0.690 | 4.101 | ≤0.001 |
| Llama December 2020 / Llama May 2021 | 5.083 | 1.605 | 5.149 | ≤0.001 |
| Llama December 2020 / Llama June 2022 | 2.773 | 0.690 | 4.101 | ≤0.001 |
| Control May 2021 / Llama May 2021 | 0.418 | 0.094 | -3.877 | 0.002 |
| Control May 2021 / Control June 2022 | 0.545 | 0.196 | -1.689 | 1 |
| Llama May 2021 / Llama June 2022 | 0.545 | 0.196 | -1.689 | 1 |
| Control June 2022 / Llama June 2022 | 0.418 | 0.094 | -3.877 | 0.002 |

**Figure S4**. Effect of (a) the 2019 initial plot cover (log-transformed) on the percentage change in cover for the Control and Llama subplots

**
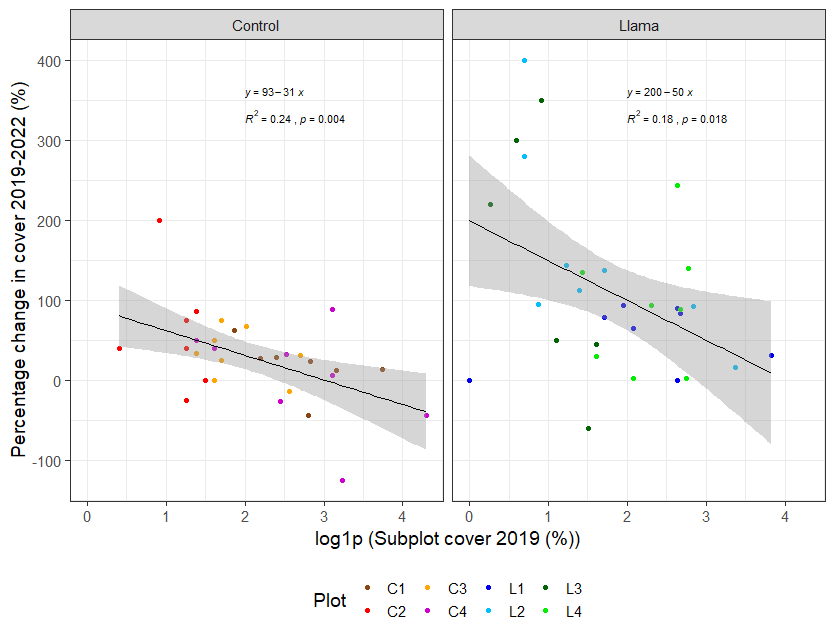
**

**Table S16.** Linear models for Plant Cover of *Cinnagrostis rigida*, *Senecio sublutescens*, *Minoides kunthiana* and *Agrostis toluscensis*, comparing the control and llama plots.


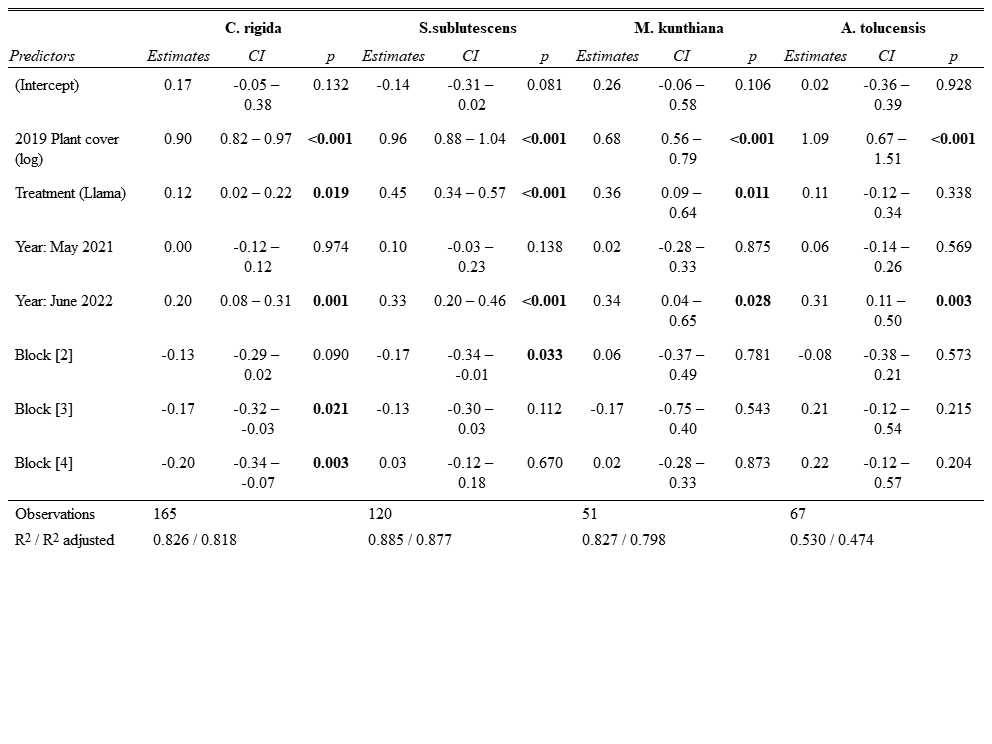


**Figure S5.** Coefficients of the models for the plant cover of the 4 most representative species.


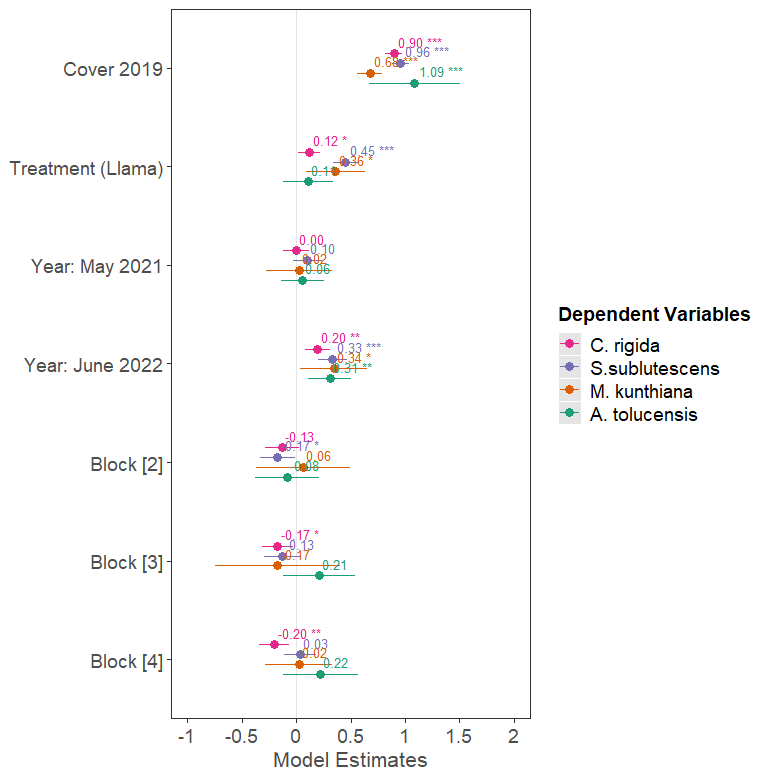


**Table S17.** Summary statistic for LDMC, Nitrogen and Phosphorus contents of *P. prostrata* leaf for the control, samples from llama plots far from dung pile (i.e., Llama: no dung pile), and samples from llama plots with proximity from dung pile (i.e., Llama: dung pile prox) treatments.

|  | **LDMC (mg.g-1)** | **Nitrogen (g.kg-1)** | **Phosphorus (g.kg-1)** |  |
| --- | --- | --- | --- | --- |
| **Pernettya prostrata** | *mean (sd)* | *mean (sd)* | *mean (sd)* | *n* |
| Control | 732.02 (57.14) | 9.32 (3.82) | 2.88 (1.11) | 31 |
| Llama: no dung pile | 760.73 (36.66) | 9.13 (3.76) | 3.27 (1.35) | 16 |
| Llama: dung pile prox | 728.76 (48.95) | 8.7 (4.66) | 3.39 (1.9) | 14 |
| **Senecio sublutescens** |  |  |  |  |
| Control | 791.42 (53.77) | 11.39 (3.43) | 3.08 (1.01) | 40 |
| Llama: no dung pile | 804.65 (39.26) | 10.91 (3.83) | 3.02 (0.99) | 20 |
| Llama: dung pile prox | 770.06 (119.63) | 12.08 (4.19) | 3 (0.75) | 20 |
| **Agrostis toluscencis** |  |  |  |  |
| Control | na | 12.44 (3.71) | 4.44 (1.47) | 40 |
| Llama: no dung pile | na | 11.65 (3.84) | 4.10 (2.14) | 19 |
| Llama: dung pile prox | na | 12.84 (3.77) | 4.32 (1.76) | 20 |
| **Cinnagrostis rigida** |  |  |  |  |
| Control | na | 9.29 (2.67) | 2.73 (0.66) | 40 |
| Llama: no dung pile | na | 8.56 (2.84) | 2.68 (0.84) | 20 |
| Llama: dung pile prox | na | 9.69 (4.1) | 2.91 (0.97) | 20 |

**Figure S6. Effect of the proximity of llama dung pile on the** LDMC of *Pernettya prostrata* (95% confidence intervals). Individuals were collected within the Control plots, within the llama plot far from dung pile influence, and with dung pile proximity. -Means not sharing any letter are significantly different by Bonferroni tests (p≤0.05). Estimates of the contrast tests are presented in **Table S19**.


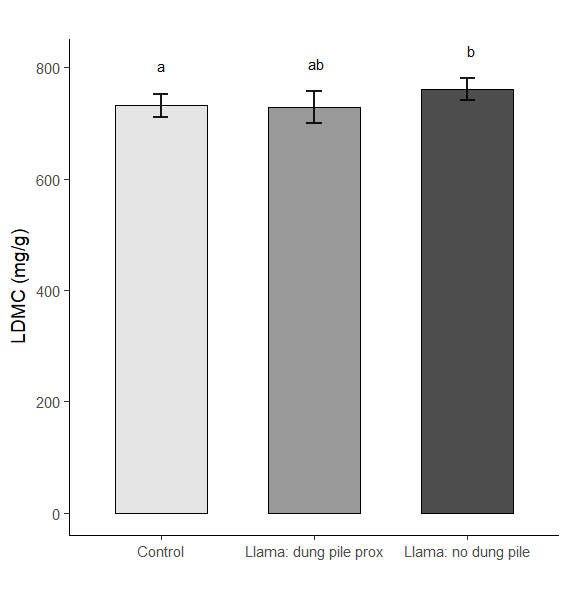


**Table S18.** Linear model of the presence of llamas on *P. prostrata* LDMC comparing control, samples from llama plots far from dung pile (i.e., Llama: no dung pile), and samples from llama plots with proximity from dung pile (i.e., Llama: dung pile prox) treatments.


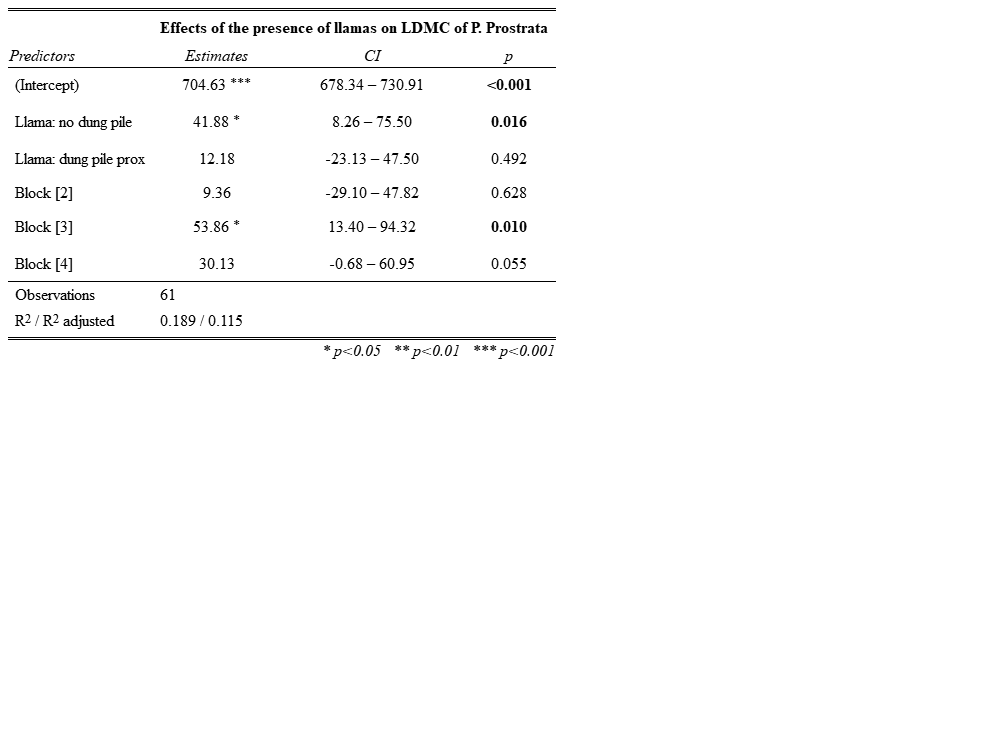


**Table S19**: Results of the contrast tests for multiple comparisons for the LDMC of *P. prostrata*. p-values adjusted with the Bonferroni procedure

| **contrast** | **estimate** | **SE** | **df** | **t.ratio** | **p.value** |
| --- | --- | --- | --- | --- | --- |
| Control - Llama: no latrine | -41.881 | 16.777 | 55 | -2.496 | **0.047** |
| Control - Llama: latrine prox | -12.183 | 17.622 | 55 | -0.691 | 1 |
| Llama: no latrine - Llama: latrine prox | 29.698 | 17.813 | 55 | 1.667 | 0.303 |

**Table S20:** Number of counts (n) of subplots with observed pasture eaten.

| **Years** | **Treatment** | **Plot** | **n** |
| --- | --- | --- | --- |
| December 2020 | Control | C3 | 1 |
|  |  | C4 | 1 |
|  | Llama | L1 | 2 |
|  |  | L2 | 1 |
|  |  | L3 | 5 |
|  |  | L4 | 5 |
| May 2021 | Control | C1 | 1 |
|  |  | C3 | 2 |
|  | Llama | L1 | 5 |
|  |  | L2 | 5 |
|  |  | L3 | 4 |
|  |  | L4 | 6 |
| June 2022 | Llama | L1 | 1 |

**Table S21:** Seed composition of the llama feces samples. The presence in the Cordillera Blanca and geographic distribution data were extracted from Smith^12^.

| **Species** | **Family** | **Genus** | **Germination viability (%)** | **Present in the experiment** | **Presence in the Cordillera Blanca** | **Genera Geographic distribution** | **Dispersal type** | **References Dispersal type** |
| --- | --- | --- | --- | --- | --- | --- | --- | --- |
| Agrostis sp. | Poaceae | Agrostis | 0 | yes | Present | Wide Temperate | Anemochory | Claure-Herrera et al., 2022 |
| Alchemilla sp. | Rosaceae | Alchemilla | 7.41 | no | Present | Wide Temperate | Epizoochory | Piiraine & Chkalov 2018 |
| Cyperaceae | Cyperaceae | na | 0 | no | Present | na | Epizoochory | Leck & Schütz 2005 |
| Cyperus sp. | Cyperaceae | Cyperus | 0 | no | Present | Wide Tropical | Hydrochory | Leck & Schütz 2005 |
| Eleocharis sp. | Cyperaceae | Eleocharis | 0 | no | Present | Cosmopolitan | Hydrochory, Epizoochory | Leck & Schütz 2005 |
| Ericaceae | Ericaceae | na | 0 | no | Present | na | Endozoochory | Claure-Herrera et al., 2022 |
| Galium sp. | Rubiaceae | Galium | 0 | no | Present | Wide Temperate | Epizoochory, Endozoochory | Taylor, 1999 |
| Luzula sp. | Juncaceae | Luzula | 42.86 | yes | Present | Wide Temperate | Autochory, Endozoochory | Claure-Herrera et al., 2022 |
| Pennisetum sp. | Poaceae | Pennisetum | 70 | no | Present | Ruderal | Epizoochory | Claure-Herrera et al., 2022 |
| Pernettya prostrata | Ericaceae | Pernettya | 13.33 | yes | Present | Austral | Endozoochory | Claure-Herrera et al., 2022 |
| Sisyrinchium sp. | Iridaceae | Sisyrinchium | 0 | no | Present | American | Autochory | Claure-Herrera et al., 2022 |
| Sporobolus sp. | Poaceae | Sporobolus | 60 | no | Present | Wide Tropical | Autochory, Endozoochory | Claure-Herrera et al., 2022 |


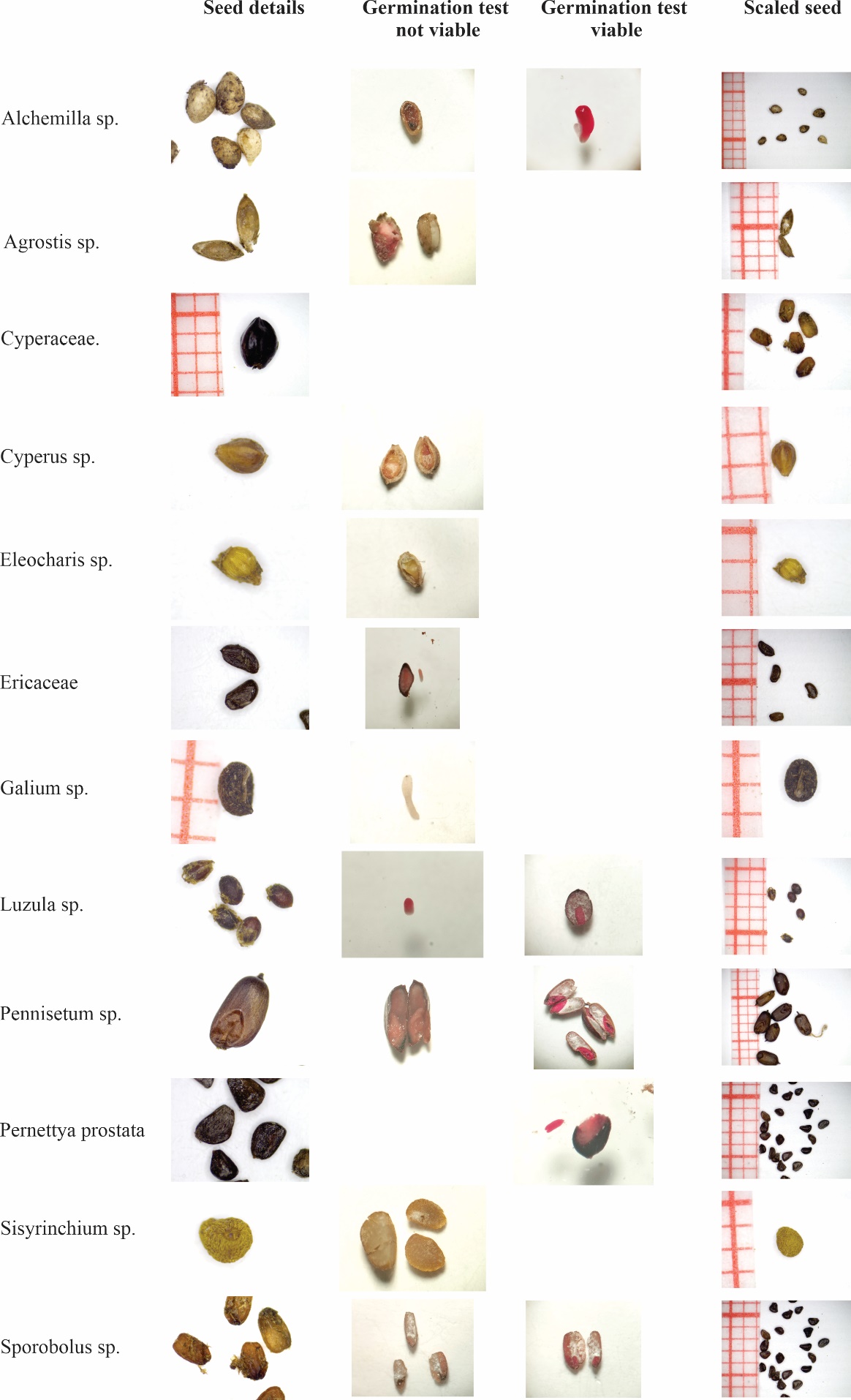
**Table S22:** Seed identification and germination test photographs

# **References**

1. Franklin, W. L. Biology, ecology, and relationship to man of the South American camelids. *Mamm. Biol. South Am.* **6**, 457–489 (1982).

2. Meneses, R. I., Loza Herrera, S., Lliully, A., Palabral, A. & Anthelme, F. Métodos para cuantificar diversidad y productividad vegetal de los bofedales frente al cambio climático. *Ecol. en Boliv.* **49**, 42–55 (2014).

3. Heiri, O., Lotter, A. F. & Lemcke, G. Loss on ignition as a method for estimating organic and carbonate content in sediments: reproducibility and comparability of results. *J. Paleolimnol.* **25**, 101–110 (2001).

4. Jensen, J. L., Christensen, B. T., Schjønning, P., Watts, C. W. & Munkholm, L. J. Converting loss‐on‐ignition to organic carbon content in arable topsoil: pitfalls and proposed procedure. *Eur. J. Soil Sci.* **69**, 604–612 (2018).

5. Matías, L., Zamora, R., Mendoza, I. & Hódar, J. A. Seed Dispersal Patterns by Large Frugivorous Mammals in a Degraded Mosaic Landscape. *Restor. Ecol.* **18**, 619–627 (2010).

6. Sayers, R. L. & Ward, R. T. Germination responses in alpine species. *Bot. Gaz.* **127**, 11–16 (1966).

7. França-Neto, J. de B. & Krzyzanowski, F. C. Tetrazolium: An important test for physiological seed quality evaluation. *J. Seed Sci.* **41**, 359–366 (2019).

8. McNeish, D. Small Sample Methods for Multilevel Modeling: A Colloquial Elucidation of REML and the Kenward-Roger Correction. *Multivariate Behav. Res.* **52**, 661–670 (2017).

9. FLorez, A., Malpartida, E. & San Martín Howard, F. Manual de forrajes para zonas áridas y semiáridas andinas. (1992).

10. Achu Nina, C. Determination of the botanical and chemical composition of the pasture diet selected by llamas (Lama glama) during the rainy season in the community of Pujrata. (2003).

11. Tapia Núñez, M. E. & Flores Ochoa, J. A. *Pastoreo y Pastizales de los Andes del Sur del Perú*. (Instituto Nacional de Investigación y Promoción Agropecuaria, Lima (Perú …, 1984).

12. Smith, D. N. *Flora and vegetation of the Huascaran National Park, Ancash, Peru, with preliminary taxonomic studies for a manual of the flora*. (Iowa State University, 1988).
